# Voice assistants in private households: a conceptual framework for future research in an interdisciplinary field

**DOI:** 10.1057/s41599-023-01615-z

**Published:** 2023-04-19

**Authors:** Bettina Minder, Patricia Wolf, Matthias Baldauf, Surabhi Verma

**Affiliations:** 1 Lucerne School of Information Technology and Computer Sciences, Lucerne University of Applied Sciences and Arts, Lucerne, Switzerland; 2 Department of Business & Management, University of Southern Denmark, Odense, Denmark; 3 Department of Management, Lucerne University of Applied Sciences and Arts, Lucerne, Switzerland; 4 Institute for Information and Process Management, Eastern Switzerland University of Applied Sciences, St.Gallen, Switzerland; 5 Department of Economics and Business Economics, Aarhus University, Aarhus, Denmark

**Keywords:** Information systems and information technology, Business and management, Science, technology and society, Criminology

## Abstract

The present study identifies, organizes, and structures the available scientific knowledge on the recent use and the prospects of Voice Assistants (VA) in private households. The systematic review of the 207 articles from the Computer, Social, and Business and Management research domains combines bibliometric with qualitative content analysis. The study contributes to earlier research by consolidating the as yet dispersed insights from scholarly research, and by conceptualizing linkages between research domains around common themes. We find that, despite advances in the technological development of VA, research largely lacks cross-fertilization between findings from the Social and Business and Management Sciences. This is needed for developing and monetizing meaningful VA use cases and solutions that match the needs of private households. Few articles show that future research is well-advised to make interdisciplinary efforts to create a common understanding from complementary findings—e.g., what necessary social, legal, functional, and technological extensions could integrate social, behavioral, and business aspects with technological development. We identify future VA-based business opportunities and propose integrated future research avenues for aligning the different disciplines’ scholarly efforts.

## Introduction

Scholarly research across disciplines agrees that technological advancement is one of the important drivers of economic development because it brings about efficiency gains for all players of an economic system (Grossman and Helpman, 1991; Kortum, 1997; Dercole et al., 2008). Digitization and emerging technologies thus usually draw intense scholarly interest and are studied with the hope that their adoption will enable companies to generate “new capabilities, new products, and new markets” (Bhat, 2005, p. 457) based on new business models, specifically designed for digitalized life spheres (Chao et al., 2007; Sestino et al., 2020; Antonopoulou and Begkos, 2020).

One of the recent emergent digital technologies promising companies substantial future revenues from innovative user services is voice assistants (VAs). They are “speech-driven interaction systems” (Ammari et al., 2019, p. 3) that offer new interaction modalities (Rzepka et al., 2022).

Partly based on the integration of complementary Artificial Intelligence (AI) technology, they allow users’ speech to be processed, interpreted, and responded to in a meaningful way. In private households, we witness a rapid adoption rate of VAs in the form of smart speakers such as Amazon Echo, Apple Homepod, and Google Home (Pridmore and Mols, 2020) which, particularly in combination with customization of IoT home systems, provide a higher level of control over the smart home experience compared to a traditional setting (Papagiannidis and Davlembayeva, 2022). Available in the United States (US) since 2014 and in Europe since September 2016 (Trenholm, 2016; Hern, 2017), by 2018, already 15.4% of the US and 5.9% of the German population owned an Amazon Echo (Brandt, 2018). Overall, private household purchases already grew to 116% in the third quarter of 2018 compared to 2017 (Tung, 2018) and, according to a recent research report from the IoT analyst firm Berg Insight (Berg Insight, 2022), the number smart homes in Europe and North America reached 105 million in 2021. We realize that, at present, VAs represent an emergent technology that has its challenges (Clark et al., 2022), similar to the Internet of Things (IoT) or big data analytics technology. It has triggered an enormous amount of diverse scholarly research resulting “in a mass of disorganized knowledge” (Sestino et al., 2020, p. 1). For both scholars and managers, the sheer quantity of disorganized information is making it hard to predict the characteristics of future technology use cases that fit users’ needs or to use this information for strategy development processes (Brem et al., 2019; Antonopoulou and Begkos, 2020). While Computer Science scholars already debate the technological feasibility of specific and complex VA applications, Social Science research points to VA-related market acceptance risks resulting, for example, from biased choices offered by VA (Rabassa et al., 2022) or from not identifying and implementing the privacy protection measures required by younger people (Shin et al., 2018), motivated by frequent user privacy leaks (Fathalizadeh et al., 2022) and worries about adverse incidents (Shank et al., 2022). Recent studies also specifically emphasize the need to shift the focus to user-centric product value (Nguyen et al., 2022) in the pursuit of the most beneficial solutions in terms of social acceptance and legal requirements (Clemente et al., 2022). For the most beneficial solutions, a collaboration between companies or even industries is likely to be necessary (Struckell et al., 2021).

There are, to our best knowledge, no systematic review papers focusing on VAs from a single discipline’s perspective that we could draw from. We did find an exploration of recent papers about the use of virtual assistants in healthcare that highlights some critical points (e.g., VA limitations concerning the ability to maintain continuity over multiple conversations (Clemente et al., 2022) or a review focusing on different interactions modalities in the ear of 4.0 industry—highlighting the need for strong voice recognition algorithm and coded voice commands (Kumar and Lee, 2022). In sum, the research that might allow for strategizing around VA solutions that match the needs of private households is scattered and needs to be organized and made sense of from an interdisciplinary perspective to shed “light on current challenges and opportunities, with the hope of informing future research and practice.” (Sestino et al., 2020). This paper thus sets out to identify, organize, and structure the available scientific knowledge on the recent use and the prospects of VAs in private households and propose integrated future research avenues for aligning the different disciplines’ scholarly efforts and leading research on consistent, interdisciplinary informed paths. We use a systematic literature review approach that combines bibliometric and qualitative content analysis to gain an overview of the still dispersed insights from scholarly research in different disciplines and to conceptualize topical links and common themes. Research on emerging technologies acknowledges that the adoption of these technologies depends on more factors than just technological maturity. Also, social aspects (e.g., social norms) and economic maturity (e.g., can a product be produced and sold so that it is cost-effective) play an important role (Birgonul and Carrasco, 2021; Xi and Hamari, 2021). Research particularly emphasizes that emerging technologies need to not only be creatively and economically explored—but also grounded in the user’s perspectives (Grossman-Kahn and Rosensweig, 2012) and serve longer enduring needs (Patnaik and Becker, 1999). IDEO conceptualized these requirements into the three dimensions of feasibility, viability, and desirability (IDEO, 2009).

Feasibility covers all aspects of VA innovation management that assures that the solution will be technically feasible and scalable. This also includes insuring that legal and regulatory requirements are met (Brenner et al., 2021). The viability lens focuses on economic success. “Desirability” ensures that the solutions and services are accepted by the target groups and, more generally, desired by society (Brenner et al., 2021). While IDEO and their focus on innovation development processes relate to a different context, the main reasoning about the relevance of these three dimensions (technical, social, and management) is also applicable when looking for research literature that helps find strategies around VA solutions that correspond to people’s needs in private homes. To cover these three dimensions, we focus on studies from Computer Science (CS), Social Science (SS), and Business and Management Science (BMS) to advance our knowledge of the still dispersed insights from scholarly research and highlight shared topics and common themes.

With this conceptual approach, we contribute an in-depth analysis and systematic overview of interdisciplinary scholarly work that allows cross-fertilization between different disciplines’ findings. Based on our findings, we develop several propositions and a framework for future research in the interest of aligning the various scholarly efforts and leading research on consistent, interdisciplinarily informed paths. This will help realize VA’s potential in people’s everyday lives. We moreover identify potential future VA-based business opportunities.

This paper is structured as follows: the section “Business opportunities related to VA use in private households” summarizes the research on potential business opportunities related to the use of VAs in private households. The research methodology, i.e., our approach of combining a bibliometric literature analysis with qualitative content analysis in a literature review, is presented in the section “Methods”. Section “Thematic clusters in recent VA research” identifies nine thematic clusters in recent VA research, and section “Analysis and conceptualization of research streams” analyzes and conceptually integrates them into four interdisciplinary research streams. Section “Discussion: Propositions and a framework for future research, and related business opportunities” identify future business opportunities and proposes future directions for integrated research, and section “Conclusion” concludes with contributions that should help both scholars and managers use this research to predict the characteristics of future technology, use cases that fit users’ needs, and use this information for their strategy development processes around VA.

## Business opportunities related to VA use in private households

Sestino et al. (2020, p. 7) argue that when new technologies emerge, “companies will need to assess the positives and negatives of adopting these technologies”. The positives of VA adoption lay mainly in the projection of large new consumer markets offering products and services where text-based human–computer interaction will be replaced by voice-activated input (Pridmore and Mols, 2020:1), checkout-free stores such as Amazon Go, and the use of VA (Batat, 2021). Marketing studies predict high adoption rates in private households due to potential efficiency gains from managing household systems and devices by voice commands anytime from anywhere (Celebre et al., 2015; Chan and Shum, 2018; Jabbar et al., 2019; Vishwakarma et al., 2019), as well as the high potential of health check app for improving communication with patients (Abdel-Basset et al., 2021) or realize self-care solutions (Clemente et al., 2022). A study by Microsoft and Bing (Olson and Kemery, 2019) substantiates that claim for smart homes by revealing that, already today, 54% of the 5000 responding US users use their smart speakers to manage their homes, especially for controlling lighting and thermostats. In surveys, users state that they envision a future in which they will increasingly use voice commands to control household appliances from the microwave to the bathtub or from curtains to toilet controls (Kunath et al., 2019). CS scholars discuss how to design complementary Internet of Things (IoT) technology features and systems to bring about such benefits (Hamill, 2006; Druga et al., 2017; Pradhan et al., 2018; Gnewuch et al., 2018; Tsiourti et al., 2018a/b; Azmandian et al., 2019; Lee et al., 2019; Pyae and Scifleet, 2019; Sanders and Martin-Hammond, 2019). BMS research additionally debates how companies should proceed to capture, organize, and analyze the (big) user data that become potentially available once VA is commonly used in private households, and to identify new business opportunities (Krotov, 2017; Sestino et al., 2020) and future VA applications, such as communication and monitoring services in pandemics (Abdel-Basset et al. 2021).

However, many recent studies also mention the negatives of VA usage, like worrying trends emerging from the so-called surveillance economy (Zuboff, 2019) or, instead, debate future questions, such as what happens when technology fails or what the rights of fully automated technological beings would be (Harwood and Eaves, 2020). 2050 out of the 5000 respondents to the Microsoft and Bing study reported concerns related to voice-enabled technology, especially about data security (52%) and passive listening (41%). The “significant new production of situated and sensitive data” (Pridmore and Mols, 2020, p. 1) in private environments and the unclear legal situation related to the usage of these data seem to act as one of the inhibitors to the adoption of more complex VA applications by users. Thus, many of the imaginable future use cases, such as advanced smart home controls (Lopatovska and Oropeza, 2018; Lopatovska et al., 2019) or personal virtual shopping assistance (Omale, 2020; Sestino et al., 2020), are still a long way off. Although technologically feasible and partly already available, today’s users use VAs for simple tasks, such as “searching for a quick fact, playing music, looking for directions, getting the news and weather” (Olson and Kemery, 2019). Therefore, companies are warned against too high expectations of fast returns. Moreover, there are also some technical issues, and only the not-yet-mature integration of further AI-enabled services in VA is expected to be a game changer leading to growth in the deployment of voice-based solutions (Gartner, 2019; Columbus, 2020).

At a meta-level, BMS research advises companies to explore and implement new technologies in their products, services, or business processes, because that might result in a considerable competitive first-mover advantage (Drucker, 1988; Porter, 1990; Carayannis and Turner, 2006; Hofmann and Orr, 2005; Bhat, 2005). At the same time, Macdonald and Jinliang (1994) have shown that in industrial gestation (or the impact of science on society), the evolution in the demand for technology, and a set of competitors go hand in hand. Consequently, the adoption of an emergent technology by “the ultimate affected customer base” (Bhat, 2005, p. 462) becomes of utmost importance when looking at how company investments pay off (Pridmore and Mols, 2020). This is particularly the case for VAs where companies are greatly dependent on the adoption of respective hardware—typically the aforementioned smart speakers (Herring and Roy, 2007)—or of new services, such as the envisioned digital assistants (Sestino et al., 2020, p. 7), by private users. VAs differ from other emergent technologies that allow companies to reap the benefits by implementing them in their own organization and reorganizing business or production processes, like RFID technology (Chao et al., 2007), nanotechnology (Bhat, 2005) or IoT-based business process (re)engineering (Sestino et al., 2020). Hence—although it is one of the most prominent emerging technologies discussed in current mass media—this might be one of the reasons for why there is yet very limited BMS research studying VA-related challenges and opportunities that could inform companies.

High-tech companies striving to develop VA-related business models need to consider and integrate scholarly knowledge from disciplines as different as CS, SS, and BMS to meet the requirements of “a secure conversational landscape where users feel safe” (Olson and Kemery, 2019, p. 24). However, such interdisciplinary perspectives are yet hardly available—instead, we see a large amount of scattered disciplinary scholarly knowledge. This situation makes it difficult to assess opportunities for future VA-related services and to develop sustainable business models that offer a potential competitive advantage. In this paper, we set out to contribute to such an assessment by organizing and making sense of the scholarly knowledge from CS, SS, and BMS. We follow earlier research on the assumption that assessing the state of emergent technologies and making sense of available knowledge on new phenomena requires an interdisciplinary perspective (Bhat, 2005; Melkers and Xiao, 2010; Sestino et al., 2020) to pin down and forecast the technology’s future impact and to advise companies in their technology adoption decisions (Leahey et al., 2017; Demidova, 2018; McLean and Osei-Frimpong, 2019). The literature review we present here is therefore additionally aimed at substantiating the call for interdisciplinarity of research into emerging technologies that aim to offer insights about business opportunities.

## Methods

**Fig. 1:**
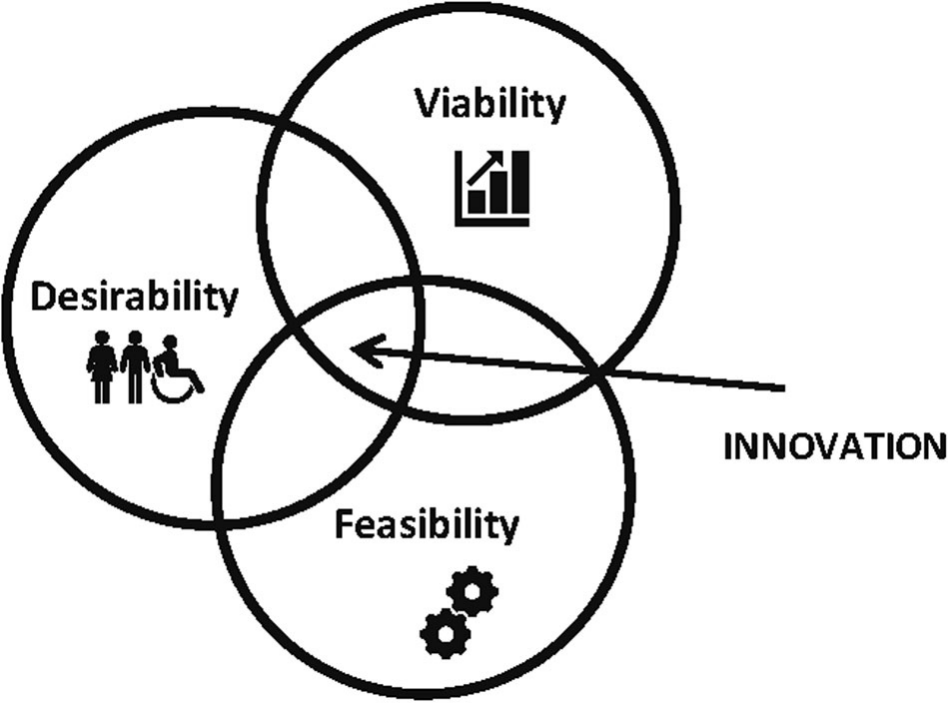
Innovation dimensions by IDEO: feasibility-viability-desirability (after IDEO, 2009).

Our aim of making sense of a large amount of disorganized scholarly knowledge on VAs, assessing challenges and opportunities for businesses, and identifying avenues for future interdisciplinary research, made a systematic literature review appear to be the most appropriate research strategy: Literature reviews enable systematic in-depth analyses about the theoretical advancement of an area (Callahan, 2014). Earlier research with similar aims that studied other emerging technologies found the method “useful for making sense of the noise” (Sestino et al., 2020:1) in a fast-growing body of scholarly literature (Fig. 1).

**Fig. 2:**
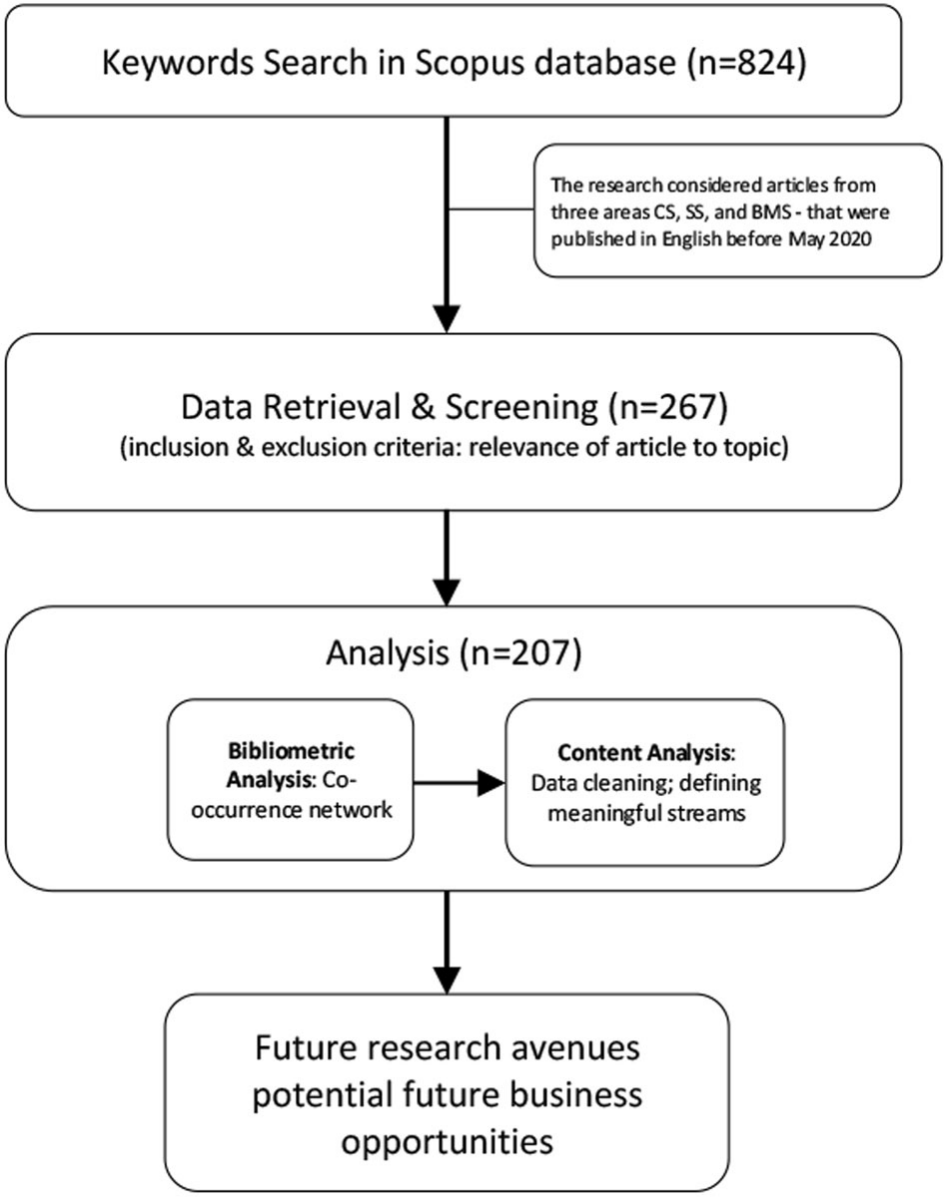
Methodological approach. Overview of the methodological research approach of this study.

For our research, we decided to combine a conventional literature review that applies qualitative content analysis, with bibliometric analysis. The bibliometric analysis provides an overview of connections between research articles and the intersection of different research areas (Singh et al., 2020). The qualitative content analysis-based literature review offers a more in-depth overview of the current state of the literature (Petticrew and Roberts, 2006). Earlier scholarly work indicates that such a combination is particularly useful for analyzing the current state of technology trends and the significance of forecasts (Chao et al., 2007; Li et al., 2019). Figure 2 depicts the methodological research approach of this study.

In the following, we describe the methodological approach in detail.

### Article identification and screening

The literature search employed the Scopus database, as the coverage for the Scopus and Web of Science databases is similar (Harzing and Alakangas, 2016). In the literature search, we employed the keywords “voice assistant” and synonyms of it (“Voice assistant” OR “Virtual assistant” OR “intelligent personal assistant” OR “voice-activated personal assistant” OR “conversational agent” OR “SIRI” OR “Alexa” OR “Google Assistant” OR “Bixby” OR “Smart Loudspeaker” OR “Echo” OR “Smart Speaker”) and “home” and synonyms of it (“home” OR “house” OR “household”). The automated bibliometric analysis scanned titles, abstracts, and keywords of the article for these terms. We used the search field “theme” including title, abstract, and keywords (compare 3.2). Due to the focus of the research, the search was restricted to articles published in the CS, SS, and BMS areas, written in English, and published before May 2020.

We adopted the Preferred Reporting Items for Systematic Reviews and Meta-Analysis (PRISMA) guide proposed by Moher et al. (2009) for the bibliometric literature review. The initial search yielded 428 articles in the CS, 356 articles in the SS, and 40 articles in BMS. After scanning the abstracts of all documents in the list of each field, further articles were excluded based on their relevance to our topic. The most frequent reason for excluding an article was that it was not about VAs—e.g., articles found with the keyword “echo” referred to acoustic phenomena. Table 1 displays the descriptive results of the bibliometric literature review.

**Table 1:** Descriptive results of the bibliometric literature review.

	Computer science	Social science	Business & management science
Total number of articles extracted	428	356	40
Total number of articles (after data cleaning)	197	52	20

*Source*: Bibliographic information obtained from Scopus on May 11, 2020.

The final dataset included 267 articles in CS, 52 articles in SS, and 20 articles in BMS.

Tables 2 and 3 display the most frequent countries of origin for SS and CS.

**Table 2:** Top countries of origin of the articles from the social science area (SS).

Country	No. of publications
United States	19
India	5
United Kingdom	5
Germany	4
Japan	4
Australia	3
Netherlands	3
Canada	2
Italy	2
Portugal	2
South Korea	2

*Source*: Bibliographic information obtained from Scopus on May 11, 2020.

**Table 3:** Top countries of origin of the articles from the computer science area (CS).

Country	No. of publications
United States	66
India	21
United Kingdom	18
Germany	15
Canada	9
Japan	9
China	8
Italy	8
Portugal	5
Singapore	5
South Korea	5

*Source*: Bibliographic information obtained from Scopus on May 11, 2020.

Tables 2 and 3 present the top countries of origin of the articles from CS and SS. There was no information related to the countries of origin of the bms articles. In view of the many (regionally differing) legal questions and regulatory issues, it is important to see that, while the US is leading the list by a large margin, the discussion is also spread over countries from different continents.

### Data analysis step 1: Bibliometric literature review

The final dataset consisted of bibliometric information including the author names, affiliations, titles, abstracts, publication dates, and citation information. The bibliometric analysis was conducted in each discipline separately using the VOSviewer software. For each discipline, we visualized common knowledge patterns through co-occurrence networks in the VA literature. A co-occurrence network contains keywords with similar meanings that can distort the analyses. Therefore, synonyms were grouped into topics using the VOSviewer thesaurus to ensure a rigorous analysis. For example, the keywords „voice assistant“, „virtual assistant“, „intelligent personal assistant“, „voice-activated personal assistant“, „conversational agent“, „SIRI“, „Alexa“, „Google Assistant“, „Bixby“, „smart loudspeaker“, „Echo“, „smart speaker“ were replaced with the main term “voice assistant”. Also, keywords were standardized to ensure uniformity and consistency (e.g., singular and plural). Further, a few keywords were also deleted from the thesaurus to ensure the focus of the review around the research questions of this study.

**Fig. 3:**
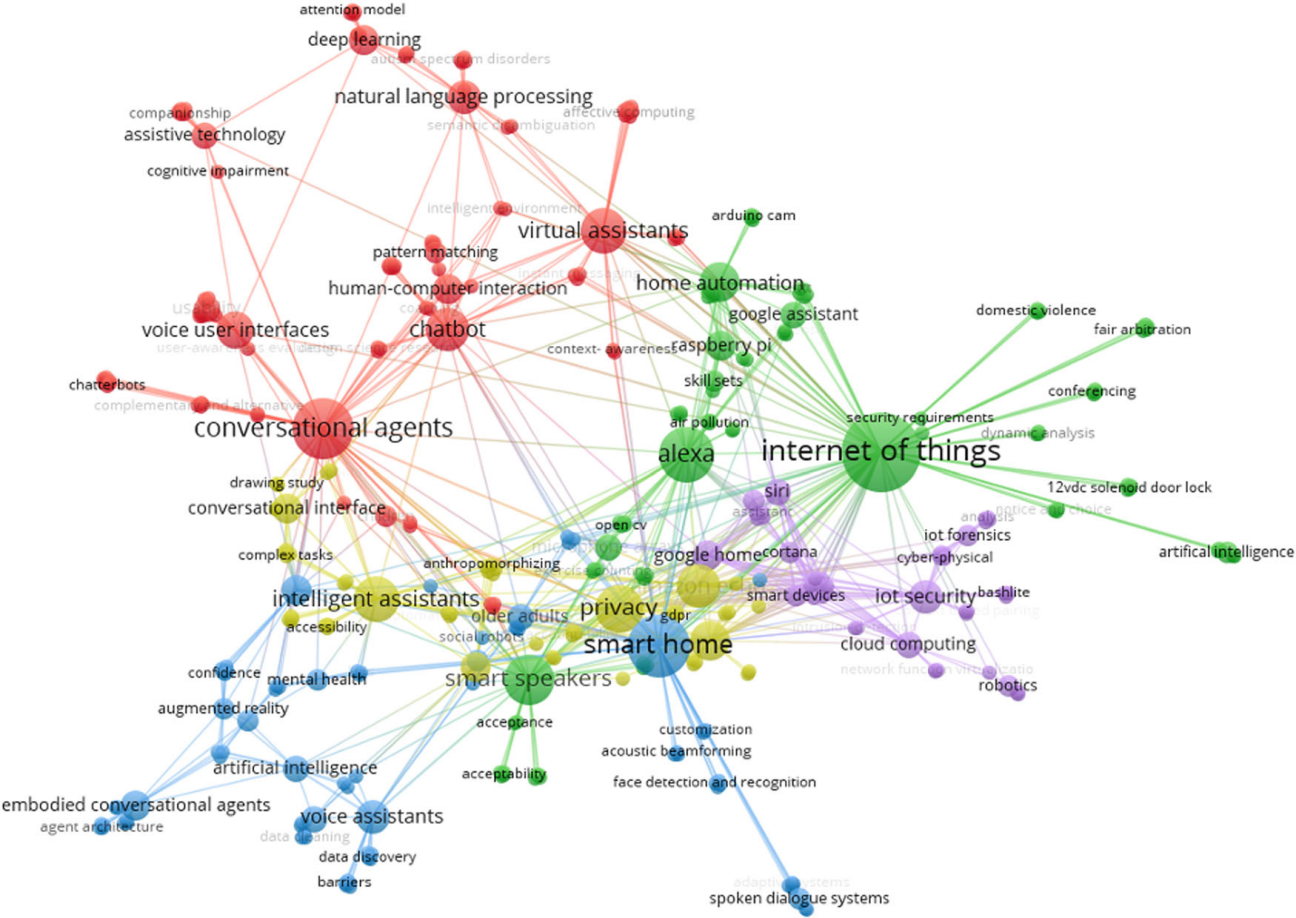
Co-occurrence network for the CS research field. The frequently co-occurring keywords, themes, or topics in research in the CS field on VAs in private households.

**Fig. 4:**
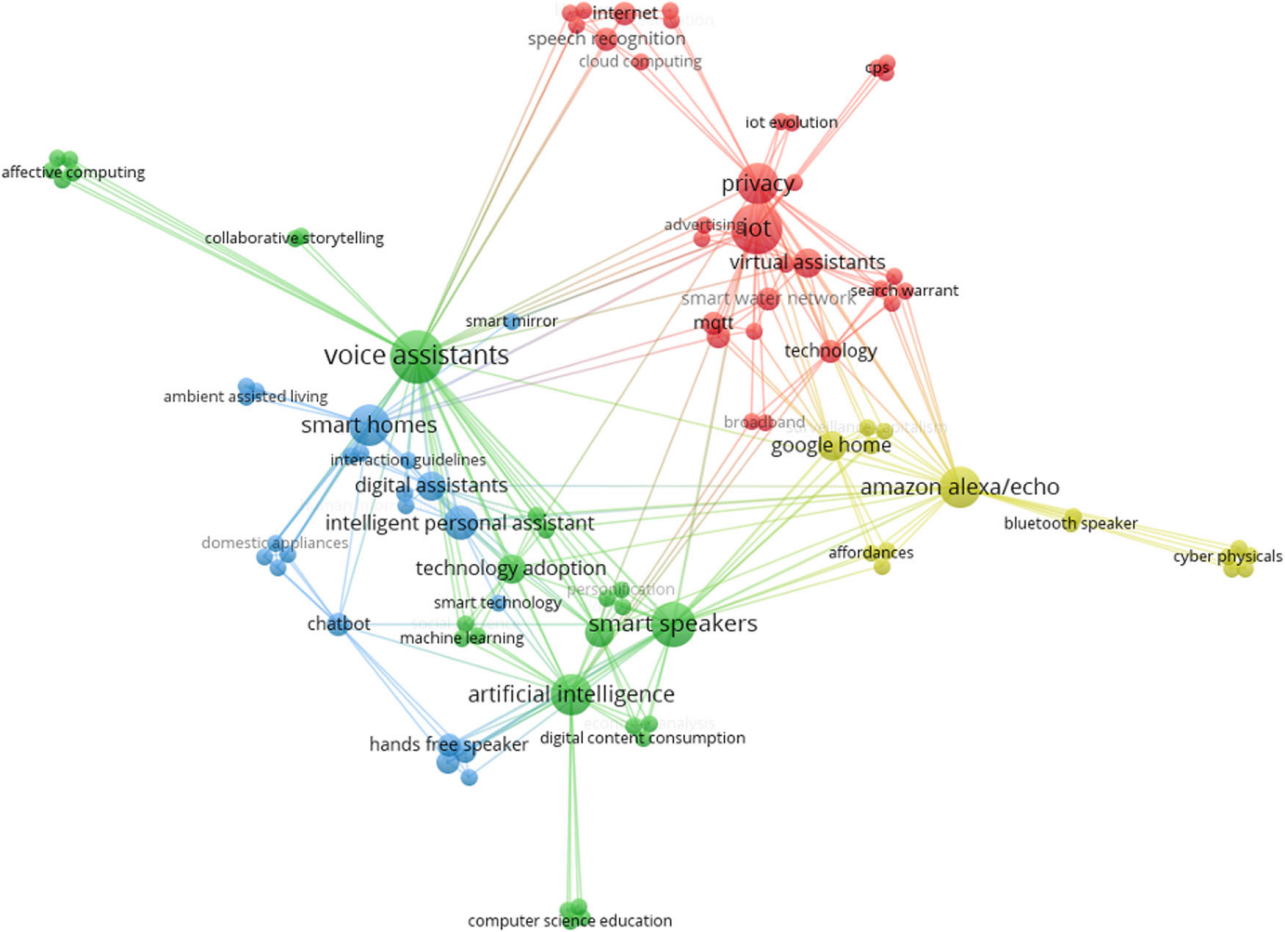
Co-occurrence network for the SS research field. The frequently co-occurring keywords, topics, or themes in research in the SS field on VAs in private households.

**Fig. 5:**
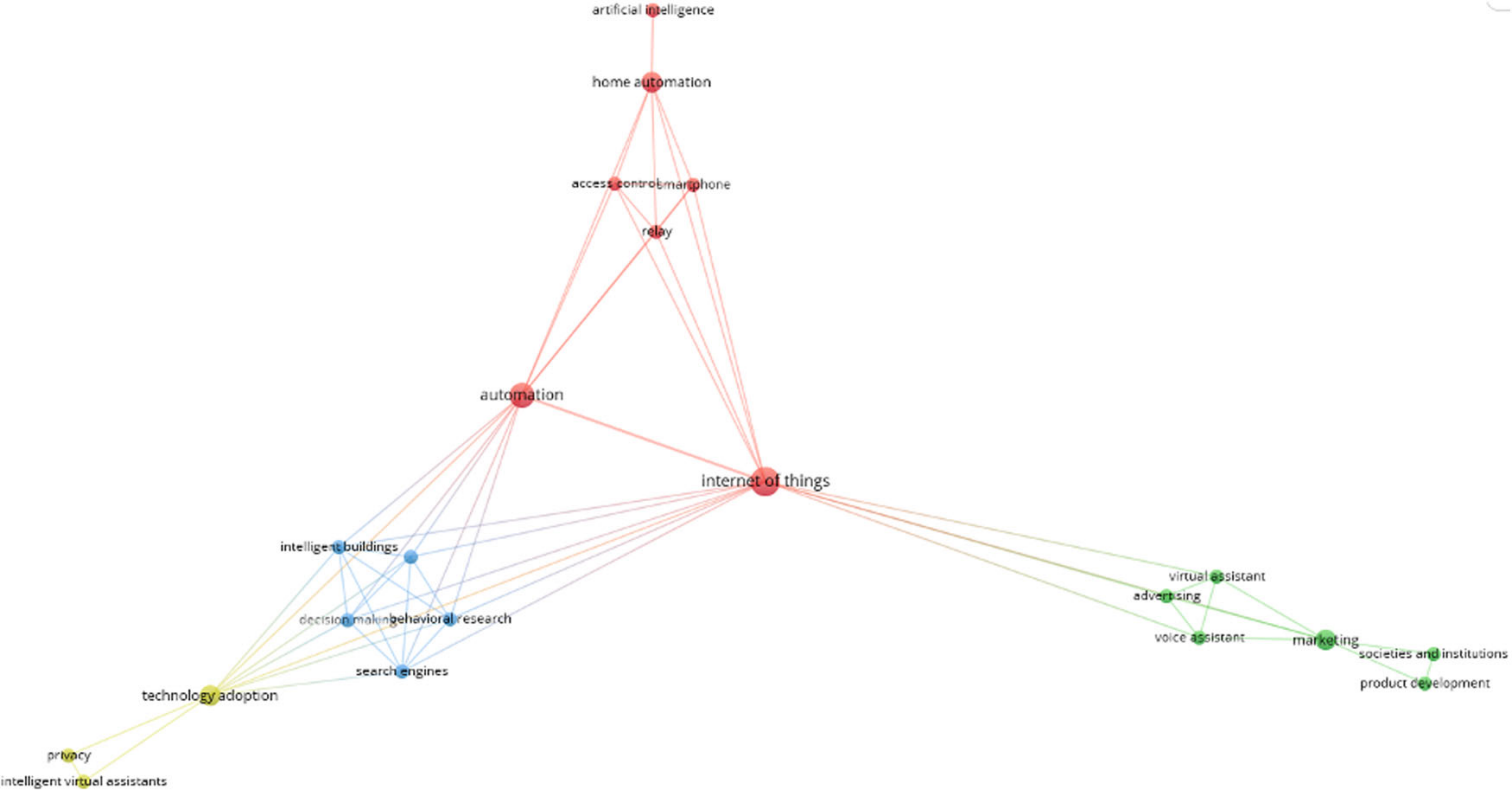
Co-occurrence network for the BMS-field. The frequently co-occurring keywords, topics, or themes in research in the BMS field on VAs in private households.

Scopus provides Subject Areas—we used these areas to generate the bibliometric analysis (e.g., select CS to analyze all papers from that area). When cleaning the data set—e.g., excluding non-relevant papers—some papers could be assigned to more than one area by checking the author’s affiliation. The co-occurrence networks (Figs. 3–5) of the keywords were obtained automatically from scanning titles, abstracts, and keywords of the articles in the final cleaned datasets. The networks present similarities between frequently co-occurring keywords (themes or topics) in the literature (Van Eck and Waltman, 2010). The co-occurrence number of two keywords is the number of articles that contain both keywords (Van Eck and Waltman, 2014). VOSviewer places these keywords in the network and identifies clusters with similar themes, and with each color representing one cluster (Van Eck and Waltman, 2010). The colors, therefore, reflect topical links and common themes. Boundaries between these clusters are fluid: ‘affordance’ for example (in Fig. 4) is in the light green cluster denoting research on VA systems—but it is also connected to the red cluster, discussing security issues (compare Fig. 4). The assignment to the ‘green cluster happens based on more frequent links to this topic. The co-occurrence networks for our three scholarly disciplines are displayed in Figs. 3–5. By discussing the clusters, nine topic themes for our research emerged (compare next section).

We can see that the networks and the topics covered differ in the three scientific areas. By studying and grouping the research topics that were revealed in the co-occurrence analysis within and across scientific areas, we identified nine thematic clusters in VA research. We labeled these clusters as “Smart devices” (cluster 1), “Human–computer interaction (HCI) and user experience (UX)” (cluster 2), “Privacy and technology adoption” (cluster 3), “VA marketing strategies” (cluster 4), “Technical challenges in VA applications development” (cluster 5), “Potential future VAs and augmented reality (AR) applications and developments” (cluster 6), “Efficiency increase by VA use” (cluster 7), “VAs providing legal evidence” (cluster 8) and “VAs supporting assisted living” (cluster 9). The clusters emerged from discussing the different research areas displayed in Figs. 3–5 in relation to our research question on strategies around VA solutions in private households. Essentially, the process of finding appropriate clusters for our research involved scanning the research areas, listening, and discussing possible grouping until the four researchers of this paper agreed on a final set of nine clusters. The nine clusters encompass different areas and terms in the figures—e.g., cluster 1 (smart devices) covered the areas ‚virtual assistants‘, ‚conversational agents‘, ‚intelligent assistants‘, ‚home automation‘, and ‚smart speakers‘, ‚smart technology‘. Cluster 2 (HCI and UX) includes areas such as ‘voice user interfaces’, ‘chatbots’, ‘human–computer interaction’, ‘hand-free speakers’. Some of the clusters we identified in this process contained only a small number of areas, such as cluster 4 (marketing strategies), which essentially covers the research areas ‘marketing’ and ‘advertising’.

### Data analysis step 2: Qualitative content analysis

It can be difficult to derive qualitative conclusions from quantitative data, which is why, in this study, we additionally conducted a qualitative content analysis of the 267 articles in the cleaned dataset. The objective of this second step was to rigorously assess the results from the bibliometric review, ensuring that the identified nine themes identified in stage 1 are in accordance with the main tenets presented in the literature. Any qualitative content analysis of literature suffers, to a certain extent, from the subjective opinions of the authors. However, the benefits of this method are indisputable and follow a well-established approach used in past studies of a similar kind. To counter the risk of subjectivity in data analysis, we involved three researchers in it, thereby triangulating investigators (Denzin, 1989; Flick, 2009). We adopted Krippendorff’s (2013) content analysis methodology to ensure a robust analysis and help with the contextual dimensions of each research field.

In the first step, the nine clusters identified by using VOSViewer were evaluated by the three researchers independently by assigning each of the 267 articles to one of the nine thematic clusters. During this process, it became apparent that the qualitative content analysis confirmed the bibliometric analysis to a large extent, i.e., that most of the articles belonged to the clusters proposed in the bibliometric analysis. However, we excluded 60 articles in this process step, since many of the less obvious thematic mismatches of the articles can only be found in a more in-depth cleaning of the data set: 5 were duplicates (4 allocated to CS, 1 to SS) and 55 papers (46 from CS, 2 from SS and 6 from BMS) were not about VAs in private households. This left us with an overall sample of 207 articles (see the list in the Appendix).

Moreover, we identified articles that belonged to other clusters than suggested in the bibliometric analysis, and assigned them, after discussions with the research team, to the correct cluster. For example, the bibliometric analyses had originally not classified any of the articles in cluster 2 (“HCI and UX”) as belonging to the BMS area, while we identified such articles during the qualitative content analysis. Table 4 below displays the distribution of articles in the final dataset.

**Table 4:** Distribution of articles in the final dataset.

Clusters	Computer Science	Social Science	Business & Management Science
1: Smart devices	Yes (23)	Yes (6)	Yes (2)
2: HCI and UX	Yes (36)	Yes (15)	Yes (5)
3: Privacy and technology adoption	Yes (28)	Yes (13)	Yes (4)
4: VA marketing strategies	No	Yes (1)	Yes (6)
5: Technical challenges in VA applications development	Yes (30)	Yes (4)	No
6: Potential future VA and AR applications and developments	Yes (35)	Yes (8)	No
7: Efficiency increase by VA use	Yes (21)B	No	Yes (3)
8: VAs providing legal evidence	Yes (3)	Yes (2)	No
9: VAs supporting assisted living	Yes (8)	Yes (5)	No
Total number of assigned articles	147	47	13

Yes = articles identified; no = no articles identified; numbers in () signify the number of identified articles.

After having accomplished this data cleaning, we developed short summary descriptions summarizing the content of the research in each of the nine clusters (see section “Thematic clusters in recent VA research”).

In a final step, we condensed the nine clusters into four meaningful streams, representing distinguishable VA research topics that can support the emergence of interdisciplinary perspectives in research that studies VAs in private households. We applied the following procedure to obtain clusters and allocate papers from the clusters to the streams: First, three researchers independently conceptualized topical research streams. Then, all researchers discussed these streams and agreed on topical headlines reflecting the terminology used in the respective research. Next, they allocated—again first working independently and later together—papers to the four research streams presented in chapter 5. Our aim of finding meaningful streams that can support the emergence of interdisciplinary research on VA in private households made a qualitative procedure appear to be the most appropriate strategy for this step in the analysis. Qualitative analysis helps organize data in meaningful units (Miles and Huberman, 1994).

## Thematic clusters in recent VA research

From our analysis, recent research on VA in private households can be divided into nine thematic clusters. In the following, we briefly present these clusters and elaborate on connections between the contributions from the three research areas we considered.

### Cluster 1: Smart device solutions

Cluster 1 comprises publications on smart device solutions in smart home settings and their potential in orchestrating various household devices (Amit et al., 2019). Many CS papers present prototypes of web-based smart home solutions that can be controlled with voice commands, like household devices enabling location-independent access to IoT-based systems (Thapliyal et al., 2018; Amit et al., 2019; Jabbar et al., 2019). A research topic that appears in both the CS and SS areas relates to users’ choices, decisions, and concerns (Pridmore and Mols, 2020). Concerns studied relate to privacy issues (Burns and Igou, 2019) or the impact of VA use on different age groups of children (Sangal and Bathla, 2019).

A topic researched in all three scientific domains is the potential of VAs for overcoming the limitations of home automation systems. CS papers typically cover suggestions for resolving mainly technical limitations, such as those concerning language options (Pyae and Scifleet, 2019), wireless transmission range (Jabbar et al., 2019), security (Thapliyal et al., 2018; Parkin et al., 2019), learning from training with humans (Demidova, 2018), or sound-based context information (Alrumayh et al., 2019). SS research mostly investigates the limitations of VAs in acting as an interlocutor and social contact for humans (Lopatovska and Oropeza, 2018; Hoy, 2018; Pridmore and Mols, 2020), or identifies requirements for more user-friendly and secure systems (Vishwakarma et al., 2019). Finally, BMS papers focus on studying efficiency gains from using VAs, for example in the context of saving energy (Vishwakarma et al., 2019).

### Cluster 2: Human–computer interaction and user experience

Cluster 2 contains human–computer interaction (HCI) research on the users’ experience of VA technology. Researchers investigate user challenges that result from unmet expectations concerning VA-enabled services (Santos-Pérez et al., 2011; Han et al., 2018; Komatsu and Sasayama, 2019). Papers from the SS area are typically discussing language issues (Principi et al., 2013; King et al., 2017).

A central topic covered both in the CS and BMS publications is trust in and user acceptance of VAs (e.g., Hamill, 2006; Hashemi et al., 2018; Lackes et al., 2019). From the BMS perspective, researchers find that trust and perceived (dis)advantages are factors influencing user decisions on buying or utilizing VAs (Lackes et al., 2019). Complementary, CS researchers find that the usefulness of human-VA interactions and access to one’s own household data impacts the acceptance of VAs (e.g., Pridmore and Mols, 2020). The combination of these two scientific disciplines discussing a topic without SS entering the debate is unique in our data material.

‘Humanized VAs’ is a topic discussed both in CS and SS research. In CS, this includes quasi-human voice-enabled assistants acting as buddies or companions for older adults living alone (Tsiourti et al., 2018a, b) or technical challenges with implementing human characteristics (Hamill, 2006; Lopatovska and Oropeza, 2018; Jacques et al., 2019). Two papers from both CS and SS contributed to the theory of anthropomorphism in the VA context (Lopatovska and Oropeza, 2018; Pradhan et al., 2019). SS additionally offers findings about user needs, like the preferred level of autonomy and anthropomorphism for VAs (Hamill, 2006).

### Cluster 3: Privacy and technology adoption

Cluster 3 consists predominantly of CS research into privacy-related aspects like the security risks of VA technology and corresponding technical solutions to minimize them (e.g., Dörner, 2017; Furey and Blue, 2018; Pradhan et al., 2019; Sudharsan et al., 2019). An exception concerns the user-perceived privacy risks and concerns that are studied in all three scientific domains. Related papers discuss these topics with a focus on user attitudes towards VA technology, resulting in technology adoption, and identify factors motivating VA application (e.g., Demidova, 2018; Fruchter and Liccardi, 2018; Lau et al., 2018; Pridmore and Mols, 2020): Perceived privacy risks are found to negatively influence user adoption rates (McLean and Osei-Frimpong, 2019). In CS studies, researchers predominantly propose solutions for more efficient VA solutions that users would want to bring into their homes (Seymour, 2018; Parkin et al., 2019; Vishwakarma et al., 2019). These should be equipped with standardized frameworks for data collection and processing (Bytes et al., 2019), or with technological countermeasures and detection features to establish IoT security and privacy protection (Stadler et al., 2012; Sudharsan et al., 2019; Javed and Rajabi, 2020). Complementary, SS researchers investigate measures for protecting the privacy of VA users beyond technical approaches, such as legislation ensuring privacy protection (Pfeifle, 2018; Dunin-Underwood, 2020).

### Cluster 4: VA marketing strategies

Cluster 4 comprises research developing strategies for advertising the use of VAs in private households. We find here articles exclusively from BMS. Scholars address various aspects of VA marketing strategies, such as highlighting security improvements or enhanced user-friendliness and intelligence of the devices (e.g., Burns and Igou, 2019; Vishwakarma et al., 2019). Others study how to measure user satisfaction with VA technology (e.g., Hashemi et al., 2018).

### Cluster 5: Technical challenges in VA applications development

Cluster 5 contains predominantly CS research papers investigating and proposing solutions for technical challenges in VA application development. Recent work focuses on extensions and improvements for the technologically relatively mature mass-market VAs (e.g., Liciotti et al., 2014; Azmandian et al., 2019; Jabbar et al., 2019; Mavropoulos et al., 2019). Some research investigates ways to overcome the technical challenges of VAs in household environments: For example, King et al. (2017) work on more robust speech recognition, and Ito (2019) proposes an audio watermarking technique to avoid the misdetection of utterances from other VAs in the same room. Further research on technological improvements includes work on knowledge graphs (Dong, 2019), on cross-lingual dialog scenarios (Liu et al., 2020), on fog computing for detailed VA data analysis (Zschörnig et al., 2019), and on the automated integration of new services based on formal specifications and error handling via follow-up questions (Stefanidi et al., 2019).

We identify a complementarity between CS and SS research within the research topic of “affective computing”. In both research domains, researchers strive to identify ways to create more empathic VAs. For example, Tao et al., (2018) propose a framework that conceptualizes several dimensions of emotion and VA use. SS research contributes to a virtual caregiver prototype aware of the patient’s emotional state (Tironi et al., 2019). However, scholarly contributions in the two areas are not related to each other.

### Cluster 6: Potential future VA applications and developments

Cluster 6 investigates the future of VAs research, particularly technological advancements we can expect and suggestions for future research avenues. Most CS papers introduce prospective potential technical applications in many different areas, such as medical treatment and therapy (Shamekhi et al., 2017; Pradhan et al., 2018; Patel and Bhalodiya, 2019) or VA content creation and retrieval (Martin, 2017; Kita et al., 2019). A sub-group of papers also proposes functional prototypes (e.g., Yaghoubzadeh et al., 2015; Freed et al., 2016; Tielman et al., 2017).

We identify three topics that are discussed in both SS and CS publications. The first focuses on language and VAs and represents an area where CS research relates to SS findings: While SS identifies open language issues in dialogs with VAs (Martin, 2017; Ong et al., 2018; Huxohl, et al., 2019), CS researchers investigate how to approach them - not only at the technological level of speech recognition but also in terms of what it means to have a conversation with a machine (Yaghoubzadeh et al., 2015; Ong et al., 2018; Santhanaraj and Barkathunissa, 2020). A second focus is on near-future use scenarios (Hoy, 2018; Seymour, 2018; Tsiourti et al., 2019; Burns and Igou, 2019) such as VA library services, VA services for assisted living or support VAs for emergency detection and handling. The third common topic is about identifying future differences between the use of VAs in private households and in other environments like public spaces (Lopatovska and Oropeza, 2018; Robinson et al., 2018).

### Cluster 7: Efficiency increase by VA use

Cluster 7 consists of papers about efficiency increase through VA use—with a focus on smart home automation systems. Papers in BMS discuss the increasing efficiency of home automation systems through the use of VAs (Vishwakarma et al., 2019). CS papers study and appraise the efficiency of home automation solutions and use cases, more efficient VA automation systems, interface device solutions (Liciotti et al., 2014; Jabbar et al., 2019; Jacques et al., 2019), effective activity assistance (Freed et al., 2016; Palumbo et al., 2016; Tielman et al., 2017), care for elderly people (Donaldson et al., 2005; Wallace and Morris, 2018; Tsiourti et al., 2019**)**, and smart assistive user interfaces and systems of the future (Shamekhi et al., 2017; Pradhan et al., 2018; Mokhtari et al., 2019). SS has not yet contributed to this cluster.

### Cluster 8: VAs providing legal evidence

Cluster 8 addresses the rather novel topic of digital forensics in papers from the CS and SS domains. The research studies how VA activities can inform court cases. Researchers investigate which information can be gathered, derived, or inferred from IoT-collected data, and what approaches and tools are available and required to analyze them (Shin et al., 2018; Yildirim et al., 2019).

### Cluster 9: VAs supporting assisted living

Cluster 9 comprises papers on VAs supporting assisted living. CS papers explore and describe technical solutions for the application of VAs in households and everyday task planning (König et al., 2016; Tsiourti et al., 2018a; Sanders and Martin-Hammond, 2019), for improving aspects of companionship (Donaldson et al., 2005), for stress management in relation to chronic pain (Shamekhi et al., 2017), and for the recognition of distress calls (Principi et al., 2013; Liciotti et al., 2014). CS scholars also study user acceptance and the usability of VA for elderly people (Kowalski et al., 2019; Purao and Meng, 2019).

CS and SS both share a research focus on VAs helping people maintain a self-determined lifestyle (Yaghoubzadeh et al., 2015; Mokhtari et al., 2019) and on their potential and limitations for home care-therapy (Lopatovska and Oropeza, 2018; Kowalski et al., 2019; Turner-Lee, 2019), but without relating findings to each other.

## Analysis and conceptualization of research streams

When comparing the bibliometric and the qualitative content analysis, the clusters found in the bibliometric analysis were confirmed to a large extent. The comparison did, however, also lead to the allocation of some articles to different areas. The content analysis particularly helped subsume the nine clusters in four principal research streams. The overview that we gained based on the four streams points to interdisciplinary research topics that need to be studied by scholars wanting to help realize VA potential through applications perceived as safe by users.

What all research domains share to a certain extent is a focus on users’ perceived privacy risks and concerns and a focus on the impact of perceived risks or concerns on the adoption of VA technology. At the same time, our findings confirm our assumption that these complementarities are generally not well used for advancing the field: In CS, researchers predominantly study future application development and technological advancements, but—except for language issues (cluster 6)—they do not relate this much to solving challenges identified in SS and BMS research. In the following, we first present an overview of the four deduced research streams and, in the next section, propositions and the conceptual model for future interdisciplinary research that we developed based on our analysis.

The four major research streams into which we consolidated the identified nine thematic clusters from our literature review are labeled as “Conceptual foundation of VA research” (stream 1), ”Systemic challenges, enabling technologies and implementation” (stream 2),” Efficiency” (stream 3) and “VA applications and (potential) use cases” (stream 4). The streams were obtained in a qualitative procedure, where three researchers conceptualized streams independently and discussed potentially meaningful streams together (compare 3.3). Table 5 provides an overview of the four main streams identified in VA literature and presents selected publications for each of the streams.

**Table 5:** Overview of the four conceptually integrated interdisciplinary VA research streams.

Streams	1. Conceptual foundation of VA research	2. Systemic challenges, enabling technologies and implementation	3. Efficiency	4. VA applications and (potential) use cases
Description	Theoretical foundations of VA research and VA design principles Understandings intelligent VA and humanized VA, and anthropomorphism in VA interaction Concepts of challenges of VA user perception, use affordance and technology adoption Security and privacy protection concepts	VA as emerging enabling technology Systemic security issues (access control, intrusion detection) UX challenges in multi-user systems Smart home customization, access (control, mobile), machine learning System set-up (e.g., raspberry pi solutions) Legal regulations, General Data Protection Regulation (GDPR), accountability	(Online) Marketing strategies, advertising VAs to private households and institutions Raising awareness on how VA can make life/home more efficient Highlighting the link between low use and perceived low benefit.	User perspectives, real-life use of VA Prototype tests with users Potential solutions for overcoming limitations of and extending existing home automation systems Opportunities of using VA in medical care, fitness and assisted living Future use cases: IOT forensics
CS articles	45	93	12	43
SS articles	30	12	2	10
BMS articles	4	4	7	2
Total^a^	79	109	21	55
Representative publications CS	Purington et al., 2017; McLean and Osei-Frimpong, 2019; Lovato et al., 2019; Lee et al., 2019; Aylett et al., 2019; McReynolds et al., 2017; Sanders and Martin-Hammond, 2019; Pyae and Joelsson, 2018; Elahi et al., 2019; Ichikawa et al., 2019	Vaca et al., 2019; Souden and Liu, 2009; Han et al., 2018; Liu et al., 2020; Oh and Kim, 2017; Tao et al., 2018; Malik et al., 2019; Samarasinghe and Mannan, 2019b; Hu et al., 2013; Pyae and Scifleet, 2019; Giorgi et al., 2019; Javed and Rajabi, 2020; Saadaoui et al., 2019; Elahi et al., 2019; Lee et al., 2009; Li et al., 2020; Robledo-Arnuncio et al., 2007; Furey and Blue, 2018	Ilievski et al., 2018; Martin, 2017; Mokhtari et al., 2019; Sanders and Martin-Hammond, 2019; Kumar, 2018; Srikanth et al., 2019	Khattar et al., 2019; Kerekešová et al., 2019; Tielman et al., 2017; Tsiourti et al., 2018a/b; Gong et al., 2018; Mavropoulos et al., 2019; Mokhtari et al., 2019; Chan and Shum, 2018; Celebre et al., 2015; Jacques et al., 2019; König et al., 2016; Shamekhi et al., 2017; Robinson et al., 2018; Masutani et al., 2019; Ong et al., 2018; Donaldson et al., 2005; Solorio et al., 2018; Calaça et al., 2019
Representative Publications SS	Pfeifle, 2018; Lopatovska et al., 2019; Martin, 2017; McLean and Osei-Frimpong, 2019; Pradhan et al., 2019; Druga et al., 2017; Brause and Blank, 2020; Dunin-Underwood, 2020; Kuruvilla, 2019	Brandt, 2018; Principi et al., 2013; Brasser et al., 2018; Samarasinghe and Mannan 2019a/b	Vora et al., 2017; Jones, 2018	Tironi, et al., 2019; Martin, 2017; Kandlhofer et al., 2016; Pradhan et al., 2018; Vora et al., 2017
Representative Publications BMS	Hamill, 2006; Kowalczuk, 2018	Goud and Sivakami, 2019; Hashemi et al., 2018	Lackes et al., 2019; Wakefield, 2019; Kowalczuk, 2018; Portillo and Lituchy, 2018; Hamill, 2006	Deshpande and Itole, 2019; Vishwakarma et al., 2019

^a^57 articles were assigned to more than one strea.

The streams systematize the scattered body of VA research in a way that offers clearly distinguishable interdisciplinary research avenues to assist in strategizing around and realizing VA technology potential with applications that are perceived as safe and make a real difference in the everyday life of users. The first stream includes all papers offering theoretical and conceptual knowledge. Papers, for example, conceptualize challenges for VA user perceptions or develop security and privacy protection concepts. Systemic challenges and enabling technologies to form a second stream in VA research. This particularly includes systemic security and UX challenges, and legal issues. Efficiency presents the third research stream, in which scholars particularly investigate private people’s awareness of how VA can make their homes more efficient and asks how VA can be advertised to private households. Finally, VA applications and potential use cases form a fourth research stream. It investigates user expectations and presents prototypes for greater VA use in future home automation systems, medical care, or IOT forensics.

The overview that we gain based on the four streams enables us to frame the contributions of the research domains to VA research more clearly than based on the nine clusters. We find that all research areas contribute publications in all streams. However, the number of contributions varies: CS acts as the main driver of current developments with most publications in all research streams. CS research predominantly addresses systemic challenges, enabling technologies and technology implementation. We recognize increasing scholarly attention on user-oriented VA applications and on VA systems for novel applications beyond their originally intended usage—such as exploiting the microphone array for sensing a user’s gestures and tracking exercises (Agarwal et al., 2018; Tsiourti et al., 2018a/b), or using VA data for forensics (Dorai et al., 2018; Shin et al., 2018)—which indicates that the fundamental technical challenges in the development of this emergent technology are solved. SS so far mainly contributed to the theoretical foundation of VA design principles and use affordance (Yusri et al., 2017), and with the theory that supports developing concrete applications. It also conceptualizes the potential or desirable impact of VA in real-life settings, such as increasing the comfort and quality of life through low-cost smart home automation systems combining VA and smartphones (Kodali et al., 2019), or VA adding to content creation (Martin, 2017). The contributions by BMS scholars are mainly aimed at researching and promoting efficiency increases from using VAs.

## Discussion: Propositions and a framework for future research, and related business opportunities

In this paper, we used a systematic literature review approach combining a bibliometric and qualitative content analysis to structure the dispersed insights from scholarly research on VAs in CS, SS, and BMS, and to conceptualize linkages and common themes between them. We identified four major research streams and specified the contributions of researchers from the different disciplines to them in a conceptual overview. Our research allows us to confirm advances in the technological foundations of VAs (Pyae and Joelsson, 2018; Lee et al., 2019; McLean and Osei-Frimpong, 2019), and some concrete VAs like Alexa, Google, and Siri have already arrived in the mass market. Still, more technologically robust and user-friendly solutions that meet their legal requirements for data security will be needed to spark broader user interest (Kuruvilla, 2019; Pridmore and Mols, 2020).

### Propositions for future research

We find that recent research from the three domains contributes to the challenges that literature identified as hindering a broader user adaption of VA in different ways, and with different foci. Table 6 summarizes the identified challenges and domain-specific research contributions.

**Table 6:** Main VA challenges and related domain-specific research contributions.

Topic	CS	SS	BMS
Resolve perceived privacy risks to spark broader user interest in complex VA solutions	x	x	x
Create comprehensible data frameworks for VA data collection and processing	x		
Develop solutions for data security	x		
Define characteristics of perceived safe VA-environments		x	
Define VA advantages beyond efficiency gains			x
Clarify benefits of accessing own data and measurements that can create user trust in VA			x
Integrate VA into complex ecosystems	x		
Create knowledge base for identifying and designing necessary changes in regulations, insurance, and real estate	x	x	x

However, to advance VA’s adoption in private households. more complex VA solutions will need to convince users that the perceived privacy risks are solved (Kowalczuk, 2018; Lackes et al., 2019). To this end, all three research domains will need to contribute: CS is required to come up with defining comprehensible frameworks for data collection and processing (Bytes et al., 2019), and solutions to ensure data safety (Mirzamohammadi et al., 2017; Sudharsan et al., 2019; Javed and Rajabi, 2020). Complementary, SS should identify the social and legal conditions which users perceive as safe environments for VA use in private households (Pfeifle, 2018; Dunin-Underwood, 2020). Finally, BMS is urged to identify user advantages that go beyond simple efficiency gains, investigate the benefits of accessing one’s own data and find metrics for user trust in technology applications (Lackes et al., 2019). Particularly, SS research is providing potentially valuable insights into users’ perceptions and use case areas such as home medical care or assisted living that would be worth to be taken into account by CS scholars developing advanced solutions, and vice versa benefit from taking available technical solutions into consideration. Similarly, BMS scholarly research exhibits a rather narrow focus on increasing the efficiency of activities by using VA applications, and on how to market these solutions to private households. CS scholars complement this focus with technical solutions aimed at increasing the efficiency of automated home systems, but the research efforts from the two domains are not well aligned. VA security-related issues and solutions, limitations of VA applications for assisted living, and effects of humanization and anthropomorphism seem to be under-investigated topics in BMS.

Thus, our first proposition reads as follows:

**Proposition 1*:*** *To advance users' adoption of complex VA applications in private households, domain-specific disciplinary efforts of CS, SS, and BMS need to be integrated by interdisciplinary research*.

Our study has shown that this is particularly important to arrive at the necessary insights into how to overcome VA security issues and VA technological development constraints CS works on and, at the same time, deal with the effects of VA humanization (SS research) and develop VA-related business opportunities (BMS research) in smart home systems, assisted living, medical home therapy, and digital forensic. Therefore, we define the following three sub-propositions:

**Proposition 1.1***: In order to realize VA potential for medical care solutions that are perceived as safe by users, research insights from studies on VA perception and on perceived security issues from SS need to be integrated with CS research aimed at resolving the technical constraints of VA applications and with BMS research about the development of use cases desirable for private households and related business models*.

**Proposition 1.2***: To advance smart home system efficiency and arrive at regulations that make users perceive the usage of more complex applications as safe*, research insights from studies on *systemic integration, and security-related technical solutions from CS need to be studied and developed*.

**Proposition 1.3***: In order to increase our knowledge of social and economic conditions for VA adoption in private households, BMS and SS research needs to integrate insights from research with users with VA prototypes and research about near-future scenarios of VA use to model and test valid business cases that are not based on mere assumptions of efficiency gains*.

In our four streams, we moreover recognize a common interest in studying VAs beyond isolated voice-enabled ‘butlers’. In essence, VAs are increasingly investigated as gateways to smart home systems which are enabling interaction with entire ecosystems. This calls, next to the development of more complex technical applications in CS, mainly for more future research into the social (SS) and economical (BMS) conditions enabling the emergence of such ecosystems—from the necessary changes in regulations to insurance and real estate issues to designing marketing strategies for VA health applications in the home (Olson and Kemery, 2019; Bhat, 2005; Melkers and Xiao, 2010; Sestino et al., 2020). The above is not only true for the three scientific domains which we looked at, but also calls for the integration of complementary VA-related research in adjacent disciplines, such as law, policy, or real estate. Our second proposition thus reads as follows:

**Proposition 2:** *To advance users’ adoption of complex VA applications in private households, research needs to perform interdisciplinary efforts to study and develop ways to overcome ecosystem-related technology adoption challenges*.

### Conceptual framework for future research

As outlined above, future research wishing to contribute to increasing user acceptance and awareness and to generate use cases that make sense for private households in everyday life is urged to make interdisciplinary efforts to integrate complementary findings.

**Fig. 6:**
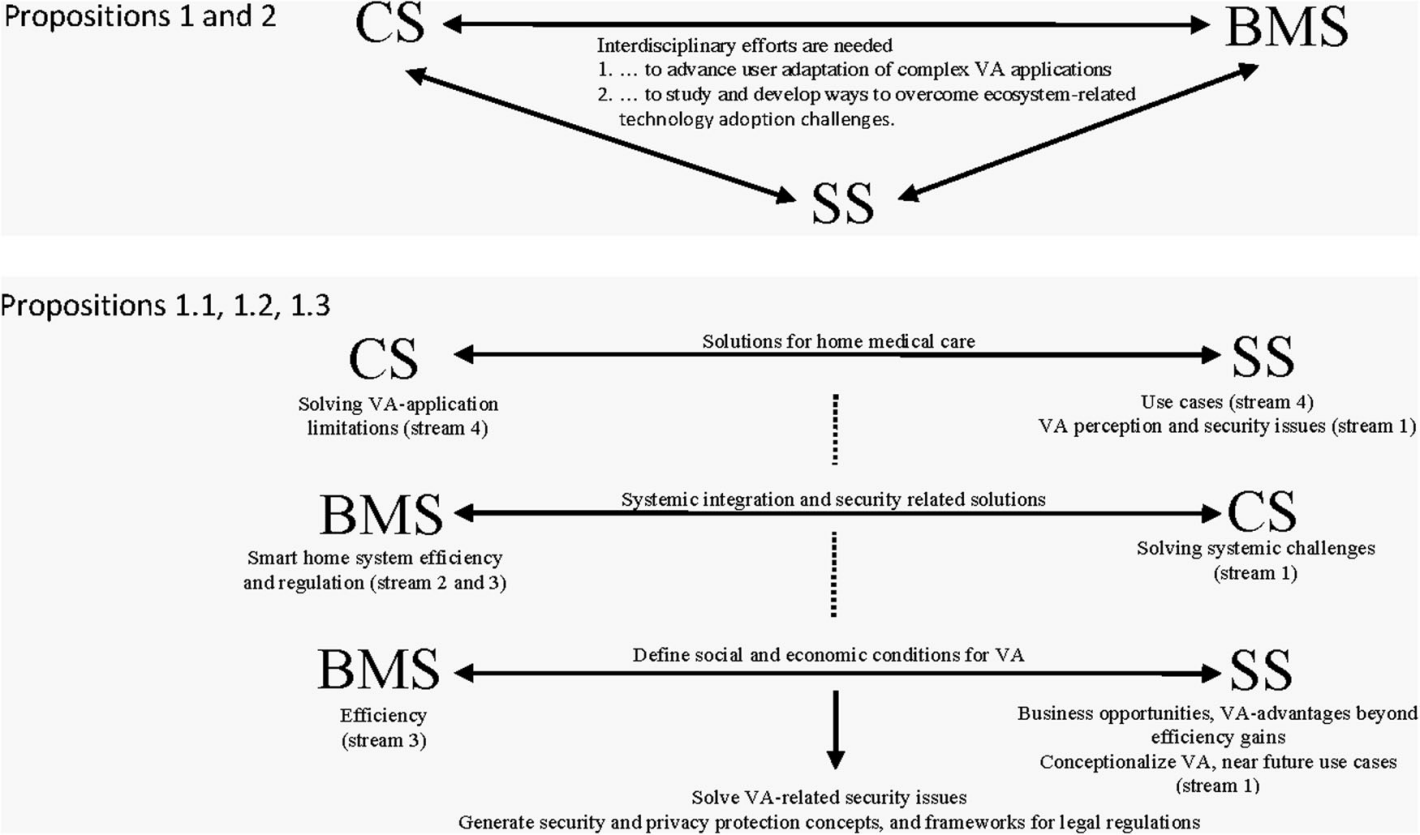
Conceptual framework for future research. The framework highlights the focus of propositions 1 and 2 and reflecting propositions 1.1, 1,2, and 1.3.

The conceptual framework (Fig. 6) presents avenues for future research. The figure highlights Propositions 1 and 2 that emphasize the need to advance user adaptation through interdisciplinary research that can help overcome challenges from complex VA applications (Proposition 1) and ecosystem-related technology adoption challenges (Proposition 2), to advance users’ adoption of complex VA applications. Furthermore, the figure reflects three sub-propositions that summarize relevant avenues for interdisciplinary work that can help solve VA-related security issues, generate security and privacy protection concepts, and advance frameworks for legal regulations. The first sub-propositions is research that helps find solutions for home medical care where VA limitations and security issues are solved. Sub-proposition 2 consists of research needed to advance systemic integration and security-related solutions for efficiency and the regulation of smart home systems. The third sub-proposition involves research that can help define social and economic conditions for VA and create business opportunities by including insights from user research with VA prototypes and from research with near-future scenarios that can model and test valid business cases that are not based on mere assumptions of efficiency gains.

### Identified business opportunities that will help realize VA potential

Overall, we confirm that VA is not a technology that enables companies to profit from implementing it in their own organizations or make business processes more efficient like other technological innovations (Bhat, 2005; Chao et al., 2007; Sestino et al., 2020). Instead, we find that companies need to build business models around VA-related products and services that users perceive as safe and beneficial. Table 7 below provides an overview of potential areas providing such business opportunities, the technology maturity of these areas, and social and business-related challenges, which need to be solved to fully access VA potential for the everyday life of users.

**Table 7:** Overview of VA business opportunity areas and disciplinary challenges.

Business opportunity area	CS challenges	SS challenges	BMS challenges
Smart home systems	Low (high technological maturity level)	High (large privacy and data safety concerns)	Medium (business models need to take into account privacy and data safety concerns)
Assisted living and medical home therapy	High (low level of technological maturity in affective computing)	High (requires understanding user needs for emotional intelligent support)	High (requires developing new business models in public-private partnerships)
Digital forensics	Medium (relatively high level of technological maturity)	High (requires defining legal frameworks)	High (requires developing new business models in public-private partnerships)

As shown, the three areas where we identified business opportunities from literature, i.e. smart home systems (Freed et al., 2016; Thapliyal et al., 2018; Jabbar et al., 2019), assisted living and medical home therapy (König et al., 2016; Tsiourti et al., 2018a/b; Sanders and Martin-Hammond, 2019), and digital forensics (Shin et al., 2018; Yildirim et al., 2019) exhibit different technology, social system conditions, and business model maturity models. It is relevant to say that, although in our review, cluster 8 ‘digital forensics’ consisted of only two papers, we can expect this to be an increasingly salient cluster in the next few years due to the importance of the topic for governmental bodies and society.

Designing appropriate business models will require companies, in the first step, to develop a deep understanding of the potential design of future ecosystems, i.e. of “the evolving set of actors, activities, and artifacts, including complementary and substitute relations, that are important for the innovative performance of an actor or a population of actors.” (Granstrand and Holgersson, 2020, p. 3). We here call for interdisciplinary research that develops and integrates the necessary insights in a thorough and, for companies, comprehensible manner.

### Methodology

In this paper, we used a relatively new approach to a literature review: We combined an automated bibliometric analysis with qualitative content analysis to gain holistic insights into a multi-faceted research topic and to structure the available body of knowledge across three scientific domains. In doing so, we followed the advice in recent research that found the classical, purely content-based literature reviews to be time-consuming, lacking rigor, and prone to be affected by the researchers’ biases (Caputo et al., 2018; Verma and Gustafsson, 2020). Overall, we can confirm that automating literature research through VOSviewer turned out to be a time-saver regarding the actual search across (partly domain-specific) sources and the collection of scientific literature, and it allowed us to relatively quickly identify meaningful research clusters based on keywords in an enormous body of data (Verma, 2017; Van Eck and Waltman, 2014). However, we also found that several additional steps were necessary to assuring the quality of the review: Despite the careful selection of keywords, the initial literature list contained several irrelevant articles (i.e., not addressing VA-related topics, yet involving the keywords ‘echo’ and ‘home’).

Thus, manual cleaning of the literature lists was required before meaningful graphs could be generated by VOSviewer. The consequent step of identifying research clusters from the graphs demanded broad topical expertise. We found this identification of clusters to be—as described by Krippendorff (2013)—a necessarily iterative process, not only to continuously refine meaningful clusters but also to reach a common understanding and interpretation in an interdisciplinary team. In a similar vein, deriving higher-level categories, i.e. the research streams, turned out to require iterative refinements.

Retrospectively, the quantitative bibliometric analysis helped in recognizing both core topics and gaps in VA-related research with comprehensive reach. The complementary content analysis yielded insights into intersections and overlaps in research by the different areas considered and enabled the identification of further promising avenues for interdisciplinary research.

## Conclusions

From our study, we conclude that research into VA-based services is not taking advantage of the potential synergies across disciplines. Business opportunities can specifically be found in spaces that require the combination of research domains that are still disconnected. This should be taken into account when looking for information that can help predict the service value of smart accommodation (Papagiannidis and Davlembayeva, 2022) or characteristics of future technology use cases that can fit users’ needs (Nguyen et al., 2022). This can also support scholars and managers in strategizing about future business opportunities (Brem et al., 2019; Antonopoulou and Begkos, 2020).

In consequence, our framework and the propositions we developed highlight the fact that more interdisciplinary research is needed and what type of research is needed to advance the development and application of VA in private households and, by implication, inform companies about future business opportunities.

The study also provides concrete future characteristics of VA use cases technology: Constant development in research on VAs, e.g., on novel devices and complementary technology like artificial intelligence and virtual reality, suggests that future VAs will no longer be limited to audio-only devices, but increasingly feature screens and built-in cameras, and offer more advanced use cases. Accordingly, embodied VAs in the form of for example social robots, require further technology advancement and integration, and studies on user perception.

### Implications for managers

Our research enabled us to identify and describe the most promising areas for business opportunities while highlighting related technological, social, and business challenges. From this, it became obvious that managers need to take all three *dimensions and related* types of challenges into account in order to successfully predict characteristics of future technology use cases that fit users’ needs, and use this information for their strategy development processes (Brem et al., 2019; Antonopouloua and Begkos, 2020). This requires not just the design of new services and business models, but of complete business ecosystems, and the establishment of partnerships from the private sector. We moreover found that establishing trust in the safe and transparent treatment of privacy and data is key in getting users to buy and use services involving VA, while pure efficiency-based arguments are not enough to dispel current worries of potential users, like the data security of technology used to improve the tracking and monitoring of patients or viruses (Abdel-Basset et al., 2021).

Although our study *investigated* VAs in private households, with the growing acceptance of working from home, not the least due to the experiences made in the COVID-19 pandemic, our findings also have implications for organizing homework environments. While, for example, the Alexa “daily check” and Apple health check app can provide a community-based AI technology that can support self-testing and virus tracking efforts (Abdel-Basset et al., 2021), managers will need to ensure that company data is safe, and this will require them to consider how their employees use VA hardware at home.

### Limitations

As with most research, this study has its limitations. While we see value in the combined approach taken in this research, as it allows insights around strategies for VA solutions that match the needs of private households, limitations can be seen in the qualitative approach of our methodology, which is subject to a certain degree of author subjectivity. Limitations of our work also relate to the fact that we included only articles from the Scopus database in this review. Thus, future research should consider articles published in other databases like EBSCO, Web of Science, or Google Scholar. Also, the study focused on only three scientific domains up to May 2020. This review paper does not offer a discussion of the consequences of the ongoing changes triggered by the Covid-19 pandemic for the use of VA solutions in private households. The impact of this disruptive pandemic experience on the use of VA is not yet well understood. More research will be necessary to obtain a complete account of how Covid-19 transformed the use of VA in private homes today and to help understand the linkages and intersections between further research areas using the same methodology.

The combined bibliometric and qualitative content analysis provided an overview of connections and intersections, and an in-depth overview of current research streams. Future research could conduct co-citation and/or bibliographic coupling analyses of authors, institutions, countries, references, etc. to complement our research.

## Supplementary information

Supplemental Material File #1

## Data Availability

Datasets were derived from public resources. Data sources for this article are provided in the Methods section of this article. Data analysis documents are not publicly available as researchers have moved on to other institutions.
