# Supplementary material for: Voice assistants in private households: a conceptual framework for future research in an interdisciplinary field
Source: Humanit Soc Sci Commun. 2023 Apr 19;10(1):173. doi: 10.1057/s41599-023-01615-z (PMC10113989; doi:10.1057/s41599-023-01615-z)
Supplement: Supplementary file 1 — Supplemental Material File #1 [file 41599_2023_1615_MOESM1_ESM.docx]

Appendix: Articles included in the literature review

**A. Computer Science domain (197)**

1. Agarwal A., Jain M., Kumar P., and Patel S. (2018). See reference list for details.
2. Alam H., Kumar A., Vyas M., Werner T., and Hartono R. (2017). Research in posterior ASR grammar modification. In H. R. Arabnia, D. de la Fuente, E. B. Kozerenko, J. A. Olivas, F. G. Tinetti (Eds.), *2017 World Congress in Computer Science, Computer Engineering and Applied Computing, CSCE 2017 – Proceedings of the 2017 International Conference on Artificial Intelligence, ICAI 2017* (pp. 350-351). CSREA Press.
3. Alias M. Y., and Kayani A. A. (2009). Effects of permittivity of insulating materials on OFDM performance in power line communications. *ICSIPA09 – 2009 IEEE International Conference on Signal and Image Processing Applications, Conference Proceedings*, 443-448. <https://doi.org/10.1109/ICSIPA.2009.5478695>
4. Al-Rawi M., Galdran A., Isasi A., Elmgren F., Carbonara G., Falotico E., Real-Arce D. A., Rodriguez J., Bastos J., and Pinto M. (2017). Cubic spline regression based enhancement of side-scan sonar imagery. *OCEANS 2017 – Aberdeen*, 1-7. <https://doi.org/10.1109/OCEANSE.2017.8084567>
5. Alrawi O., Lever C., Antonakakis M., and Monrose F. (2019). SoK: Security evaluation of home-based IoT deployments. *Proceedings – IEEE Symposium on Security and Privacy*, May-2019, 1362-1380. <https://doi.org/10.1109/SP.2019.00013>
6. Alrumayh A. S., Lehman S. M., and Tan C. C. (2019). See reference list for details.
7. Amit S., Koshy A. S., Samprita S., Joshi S., and Ranjitha N. (2019). See reference list for details.
8. Angelini L., Caon M., Casas J., Cena F., Rapp A., Khaled O.A., and Mugellini E. (2018). Ubiquitous chatbots: Workshop on wearable and embodied conversational agents. *UbiComp/ISWC 2018 – Adjunct Proceedings of the 2018 ACM International Joint Conference on Pervasive and Ubiquitous Computing and Proceedings of the 2018 ACM International Symposium on Wearable Computers,* 1652-1655. <https://doi.org/10.1145/3267305.3274146>
9. Aruna T.M., Satyanarayana M.S., and Divyaraj G.N. (2019). A unique work of out of sight epigraphy creation for data security. *Journal of Advanced Research in Dynamical and Control Systems, 11*(7), pp. 308-311. Institute of Advanced Scientific Research.
10. Aylett M.P., Clark L., and Cowan B.R. (2019). See reference list for details.
11. Azmandian M., Arroyo-Palacios J., and Osman S. (2019). See reference list for details.
12. Bhalla A. (2018). An exploratory study understanding the appropriated use of voice-based search and assistants. *ACM International Conference Proceeding Series*, 90-94. <https://doi.org/10.1145/3297121.3297136>.
13. Braiek H. B., and Khomh F. (2020). On testing machine learning programs. *Journal of Systems and Software*, *164*. <https://doi.org/10.1016/j.jss.2020.110542>.
14. Buchner H., and Kellermann W. (2008). A fundamental relation between blind and supervised adaptive filtering illustrated for blind source separation and acoustic echo cancellation. *2008 Hands-Free Speech Communication and Microphone Arrays, Proceedings*, 17–20. <https://doi.org/https://doi.org/10.1109/HSCMA.2008.4538677>.
15. Burns M. B., and Igou A. (2019). See reference list for details.
16. Bychkov A. S., Novotna V., Shevchenko V. I., and Shevchenko A. V. (2019). Improvement of the model of computer epidemics based on expanding the set of possible states of the information systems objects. *Journal of Automation and Information Sciences, 51*(11), 34-49. <https://doi.org/10.1615/JAutomatInfScien.v51.i11.40>.
17. Bytes A., Adepu S., and Zhou J. (2019). See reference list for details.
18. Calaça J., Nobrega L., and Baras K. (2019). See reference list for details.
19. Cavazza M., Smith C., Charlton D., Zhang L., Turunen M., and Hakulinen J. (2008). A ‘companion’ ECA with planning and activity modelling. *Proceedings of the International Joint Conference on Autonomous Agents and Multiagent Systems, AAMAS*, *(3),* 1257-1260. IFAAMAS.
20. Celebre A. M.D., Dubouzet A. Z. D., Medina I. B. A., Surposa A. N. M., and Gustilo R. C. (2015). See reference list for details.
21. Cenni F., Schless S.-H., Bar-On L., Aertbeliën E., Bruyninckx H., Hanssen B., and Desloovere K. (2018). Reliability of a clinical 3D freehand ultrasound technique: Analyses on healthy and pathological muscles. *Computer Methods and Programs in Biomedicine, (156*), 97-103. <https://doi.org/10.1016/j.cmpb.2017.12.023>.
22. Cha Y., Hong Y., Jang J., and Yi M. Y. (2019). “Jack-of-all-trades”: A thematic analysis of conversational agents in multi-device collaboration contexts. *Conference on Human Factors in Computing Systems – Proceedings*. Article 3313045. <https://doi.org/10.1145/3290607.3313045>.
23. Chan Z. Y., and Shum P. (2018). See reference list for details.
24. Chen P., Zhao J., and Song Y. (2018). Analysis of fog suppression effect of FMCW laser detection baseline. In Y. Huang (Ed.). *Proceedings of SPIE – The International Society for Optical Engineering*, (10964). <https://doi.org/10.1117/12.2505951>.
25. Chilcañán D., Navas P., and Escobar M. (2018). Virtual assistant for IoT process management, using a middleware. *ACM International Conference Proceeding Series*, 209-213. <https://doi.org/10.1145/3242840.3242875>.
26. Chuklin A., Severyn A., Trippas J. R., Alfonseca E., Silen H., and Spina D. (2019). Using audio transformations to improve comprehension in voice question answering. In F. Crestani, M. Braschler, J. Savoy, A. Rauber, H.Muller, D. E. Losada, G. Heinatz Burki, L. Cappellato, and N. Ferro (Eds.), *Lecture Notes in Computer Science (including subseries Lecture Notes in Artificial Intelligence and Lecture Notes in* *Bioinformatics)*. Article 11696 LNCS (pp. 164-170). Springer. <https://doi.org/10.1007/978-3-030-28577-7_12>.
27. Cicció J.A., and Quesada L. (2018). Framework for creating audio games for intelligent personal assistants. In T. Ahram and C. Falcão (Eds.), *Advances in Human Factors in Wearable Technologies and Game Design* (Vol. 608, pp. 204–214). Springer. <https://doi.org/10.1007/978-3-319-60639-2_21>.
28. Crisostomi E., Gallicchio C., Micheli A., Raugi M., and Tucci M. (2015). Prediction of the Italian electricity price for smart grid applications. *Neurocomputing, 170*, 286-295. <https://doi.org/10.1016/j.neucom.2015.02.089>.
29. Demidova E. (2018). See reference list for details.
30. Devakunchari R., Agarwal R., and Agarwal E. (2019). A survey of chatbot design techniques*. International Journal of Engineering and Advanced Technology, 8*(2), 35-39.
31. Diehl C., Schiffhauer B., Eyssel F., Achenbach J., Klett S., Botsch M., and Kopp S. (2017). Get one or create one: The impact of graded involvement in a selection procedure for a virtual agent on satisfaction and suitability ratings. In C. O’Sullivan, G. Castellano, S. Kopp, J. Beskow, C. Peters and I. Leite (Eds.), *Lecture Notes in Computer Science (including subseries Lecture Notes in Artificial Intelligence and Lecture Notes in Bioinformatics)*, Article 10498 LNAI, 109-118. Springer. <https://doi.org/10.1007/978-3-319-67401-8_13>.
32. Donaldson J., Evnin J., and Saxena S. (2005). See reference list for details.
33. Dong X.L. (2019). See reference list for details.
34. Dorai G., Houshmand S., and Baggili I. (2018). See reference list for details.
35. Dörner R. (2017). See reference list for details.
36. Druga S., Breazeal C., Williams R., and Resnick M. (2017). See reference list for details.
37. Ehrenbrink P., Hillmann S., Weiss B., and Möller S. (2016). Psychological reactance in HCI – A method towards improving acceptance of devices and services. In C. Parker (Ed.). *Proceedings of the 28^th^ Australian Computer-Human Interaction Conference, OzCHI 2016*, 478-482. Association for Computing Machinery. <https://doi.org/10.1145/3010915.3010978>.
38. Elahi H., Wang G., Peng T., and Chen J. (2019). See reference list for details.
39. 11^th^ International Conference on Cross-Cultural Design, CCD 2019, held as part of the 21^st^ International Conference on Human-Computer Interaction, HCI International 2019. (2019). In P.-L.P. Rau (Ed.), *Lecture Notes in Computer Science (including subseries Lecture Notes in Artificial Intelligence and Lecture Notes in Bioinformatics).* Springer.
40. Filimon M., Iftene A., and Trandabăt D. (2019). Bob – A general culture game with voice interaction. In I. J. Rudas, C. Janos, C. Toro, J. Botzheim, R. J. Howlett and L. C. Jain (Eds.), *Procedia Computer Science, 159*, 323-332. Elsevier. <https://doi.org/10.1016/j.procs.2019.09.187>.
41. Freed M., Burns B., Heller A., Sanchez D., and Beaumont-Bowman S. (2016). See reference list for details.
42. Fruchter N., and Liccardi I. (2018). See reference list for details.
43. Furey E., and Blue J. (2018). See reference list for details.
44. Gaina R.D., Lucas S.M., and Perez-Liebana D. (2019). Project thyia: A forever gameplayer. *IEEE Conference on Computatonal Intelligence and Games, CIG*, 2019-August. <https://doi.org/10.1109/CIG.2019.8848047>.
45. Ghazal M., Basmaji T., Qasymeh M., Salim R., and Khalil A. (2019). Localized assistive scene understanding using deep learning and the IoT. In M. Younas, I. Awan and F. Portela (Eds.), *Proceedings – 2019 International Conference on Future Internet of Things and Cloud Workshops, FiCloudW 2019,* 53-58. IEEE. <https://doi.org/10.1109/FiCloudW.2019.00023>.
46. Gill E.W., and Huang W. (2015). A review of the continuing evolution of coastal ocean radar remote sensing in the Eastern Canadian context. *2014 Oceans – St. John’s, OCEANS 2014.* <https://doi.org/10.1109/OCEANS.2014.7003223>.
47. Giorgi R., Bettin N., Ermini S., Montefoschi F., and Rizzo A. (2019). See reference list for details.
48. Gnewuch U., Morana S., Heckmann C., and Maedche A. (2018). See reference list for details.
49. Godwin S., Glendenning B., and Gagneja K. (2019). Future security of smart speaker and IoT smart home devices. In S. Piramuthu and P. Urien (Eds.), *2019 5^th^ International Conference on Mobile and Secure Services, MOBISECSERV 2019.* IEEE. <https://doi.org/10.1109/MOBISECSERV.2019.8686545>.
50. Gong Y., Yatawatte H., Poellabauer C., Schneider S., and Latham S. (2018). See reference list for details.
51. Griol D., and Molina J.M. (2011). Context-aware conversational agents using POMDPs and agenda-based simulation. In J. M. Molina, J. R. C. Corredera, A. M. B. Barbolla, M. F. C. Perez and J. Ortega-Garcia (Eds.), *Advances in Intelligent and Soft Computing, 94*, 29-36. <https://doi.org/10.1007/978-3-642-19908-0_4>.
52. Guamán S., Tapia F., Yoo S.G., Calvopiña A., and Orta P. (2018). Device control system for a smart home using voice commands: A practical case. *ACM International Conference Proceeding Series*, 86-89. <https://doi.org/10.1145/3285957.3285977>.
53. Hamill L. (2006). See reference list for details.
54. Han J., Chung A.J., Sinha M.K., Harishankar M., Pan S., Noh H.Y., Zhang P., and Tague P. (2018). See reference list for details.
55. Han X., Yeh T. (2019). Evaluating voice applications by user-aware design guidelines using an automatic voice crawler. In N. Riche, D. Parra and C. Trattner (Eds.), *CEUR Workshop Proceedings*, *2327.* CEUR-WS.
56. Hartholt A., Fast E., Reilly A., Whitcup W., Liewer M., and Mozgai S. (2019). Ubiquitous virtual humans: A multi-platform framework for embodied AI agents in XR. *Proceedings – 2019 IEEE International Conference on Artificial Intelligence and Virtual Reality, AIVR 2019*, 308-312. <https://doi.org/10.1109/AIVR46125.2019.00072>.
57. Heymann J., Bacchiani M., and Sainath T.N. (2018). Performance of mask based statistical beamforming in a smart home scenario. *ICASSP, IEEE International Conference on Acoustics, Speech and Signal Processing – Proceedings*, *2018-April,* 6722-6726. <https://doi.org/10.1109/ICASSP.2018.8462372>.
58. Hu J., Tu X., Zhu G., Li Y., and Zhou Z. (2013). See reference list for details.
59. Hu Z., Tarakji A.B., Raheja V., Phillips C., Wang T., and Mohomed I. (2019). DeepHome: Distributed inference with heterogeneous devices in the edge. *EMDL 2019 – Proceedings of the 3^rd^ International Workshop on Deep Learning for Mobile Systems and Applications, co-located with MobiSys 2019,* 13-18. <https://doi.org/10.1145/3325413.3329787>.
60. Ichikawa J., Mitsukuni K., Hori Y., Ikeno Y., Alexandre L., Kawamoto T., and Nishizaki Y., Oka N. (2019). See reference list for details.
61. Ignatenko Y. V., Tryapitsyn V. N., and Ignatenko I. Y. (2004). Comparison of timing devices on the earth and the satellite by laser ranging method*. Journal of Automation and Information Sciences, 36*(6), 44-49. <https://doi.org/10.1615/JAutomatInfScien.v36.i6.50>.
62. Ilievski A., Dojchinovski D., Ackovska N., and Kirandziska V. (2018). See reference list for details.
63. Ilievski A., Dojchinovski D., and Gusev M. (2019). Interactive voice assisted home healthcare systems. *Proceedings of the 9th Balkan Conference on Informatics*, 1–5. <https://doi.org/10.1145/3351556.3351572>.
64. International Archives of the Photogrammetry, Remote Sensing and Spatial Information Sciences (2017). In C. Ouimet, S. Fai, J. Hayes, L. Smith and M. Santana Quintero (Eds.), *International Archives of the Photogrammetry, Remote Sensing and Spatial Information Sciences – ISPRS Archives 42(2W5) C.* International Society for Photogrammetry and Remote Sensing.
65. Ito A. (2019). See reference list for details.
66. Jabbar W. A., Kian T. K., Ramli R. M., Zubir S. N., Zamrizaman N. S. M., and Balfaqih M., Shepelev V., and Alharbi S. (2019). See reference list for details.
67. Jacques R., Gerber E., Luger E., Wang D., Fslstad A., Grudin J., and Monroy-Hernández A. (2019). See reference list for details.
68. Javed Y., and Rajabi N. (2020). See reference list for details.
69. Javed Y., Sethi S., and Jadoun A. (2019). Alexa’s voice recording behavior: A survey of user understanding and awareness. *Proceedings of the 14th International Conference on Availability, Reliability and Security*, 1–10. <https://doi.org/10.1145/3339252.3340330>.
70. Kandlhofer M., Steinbauer G., Hirschmugl-Gaisch S., and Huber P. (2016). See reference list for details.
71. Karthikeyan M., Subashini T. S., and Prashanth M. S. (2020). Implementation of Home Automation Using Voice Commands. In K. S. Raju, R. Senkerik, S. P. Lanka and V. Rajagopal (Eds.), *Advances in Intelligent Systems and Computing, 1079*, (pp. 155-162). Springer. <https://doi.org/10.1007/978-981-15-1097-7_13>.
72. Kayani A. B. A., and Alias M. Y. B. (2009). Performance of OFDM in the variation of the branch lengths in power line communications. *Proceedings – MICC 2009: 2009 IEEE 9^th^ Malaysia International Conference on Communications with a Special Workshop on Digital TV Contents*, 462-467. <https://doi.org/10.1109/MICC.2009.5431552>.
73. Kennedy S., Li H., Wang C., Liu H., Wang B., and Sun W. (2019). I can hear your Alexa: Voice command fingerprinting on smart home speakers. *2019 IEEE Conference on Communications and Network Security, CNS 2019*, 232-240. <https://doi.org/10.1109/CNS.2019.8802686>.
74. Kepuska V., and Bohouta G. (2018). Next-generation of virtual personal assistants (Microsoft Cortana, Apple Siri, Amazon Alexa and Google Home). In S. Chakrabarti and H. N. Saha (Eds.), *2018 IEEE 8^th^ Annual Computing and Communication Workshop and Conference, CCWC 2018*, *2018-January,* (pp. 99-103). IEEE. <https://doi.org/10.1109/CCWC.2018.8301638>.
75. Kerekešová, V., Babič, F., and Gašpar, V. (2019). See reference list for details.
76. Khattar S., Sachdeva A., Kumar R., and Gupta R. (2019). See reference list for details.
77. Khin N. N., and Soe K. M. (2020). University Chatbot using Artificial Intelligence Markup Language. *2020 IEEE Conference on Computer Applications.* Article 9022814. <https://doi.org/10.1109/ICCA49400.2020.9022814>.
78. Kim D., Ahn D., Chai and Y. H. (2019). Real-time architectural visualization for the changing demands of Korean housing. *International Journal of Advanced Science and Technology, 28*(4), 229-238. Science and Engineering Research Support Society.
79. Kim K., Boelling L., Haesler S., Bailenson J., Bruder G., and Welch G. F. (2019). Does a digital assistant need a body? The influence of visual embodiment and social behavior on the perception of intelligent virtual agents in AR. *2018 IEEE International Symposium on Mixed and Augmented Reality (ISMAR)*, 105–114. <https://doi.org/10.1109/ISMAR.2018.00039>.
80. King B., Chen I.-F., Vaizman Y., Liu Y., Maas R., Parthasarathi S.H.K., and Hoffmeister B. (2017). See reference list for details.
81. Kloeckner K., Davis J., Fuller N.C., Lanfranchi G., Pappe S., Paradkar A., Shwartz L., Surendra M., and Wiesmann D. (2018). *Conversational IT service management, In Transforming the IT Services Lifecycle with AI Technologies* (pp. 75-93). Springer. <https://doi.org/10.1007/978-3-319-94048-9_5>.
82. Komatsu S., and Sasayama M. (2019). See reference list for details.
83. Komori M., Fujimoto Y., Xu J., Tasaka K., Yanagihara H., and Fujita K. (2019). Experimental study on estimation of opportune moments for proactive voice information service based on activity transition for people living alone. In M. Kurosu (Ed.), *Human-Computer Interaction. Perspectives on Design* (pp. 527–539). Springer. <https://doi.org/10.1007/978-3-030-22646-6_39>.
84. König A., Malhotra A., Francis L. E., and Hoey J. (2016). See reference list for details.
85. Kowalski J., Skorupska K., Kopeć W., Jaskulska A., Abramczuk K., Biele C., and Marasek K. (2019). See reference list for details.
86. Kühnel C., Weiss B., and Möller S. (2009). Talking heads for interacting with spoken dialog smart-home systems. *Proceedings of the Annual Conference of the International Speech Communication Association, INTERSPEECH*, 304-307.
87. Kumar A. (2018). See reference list for details.
88. Lackes R., Siepermann M., and Vetter G. (2019). See reference list for details.
89. Lafia S., Xiao J., Hervey T., Kuhn W. (2019). Talk of the town: Discovering open public data via voice assistants. In S. Timpf, C. Schlieder, M. Kattenbeck, B. Ludwig and K. Stewart (Eds.), *Leibniz International Proceedings in Informatics*, *142*. Dagstuhl Publishing. <https://doi.org/10.4230/LIPIcs.COSIT.2019.10>.
90. Lai Y.-C., Jhan J.-D., Yang W.-C., Kuo F.-H., and Shih T.-C. (2019). Quality of Service Measurement Mechanism of Cloud-Based Network Architecture. *2019 20^th^ Asia-Pacific Network Operations and Management Symposium: Management in a Cyber-Physical World,* Article 8893126. <https://doi.org/10.23919/APNOMS.2019.8893126>.
91. Landhäußer M., Weigelt S., and Tichy W. F. (2017). NLCI: A natural language command interpreter. *Automated Software Engineering*, *24*(4), 839–861. <https://doi.org/10.1007/s10515-016-0202-1>.
92. Lee H.-W. (2016). Stereo acoustic echo canceller using simplified orthogonal projection algorithm. *Far East Journal of Electronics and Communications, 16*(3), 527-544. <https://doi.org/10.17654/EC016030527>.
93. Lee I., Kinney C.E., Lee B., and Kalker A. A. C. M. (2009). See reference list for details.
94. Lee S., Kim S., and Lee S. (2019). See reference list for details.
95. Lei X., Tu G.-H., Liu A.X., and Li C.-Y., and Xie T. (2018). The insecurity of home digital voice assistants – Vulnerabilities, attacks and countermeasures*. 2018 IEEE Conference on Communications and Network Security*, Article 8433167. <https://doi.org/10.1109/CNS.2018.8433167>.
96. Leybourne B. (2018). Why inter-disciplinary research, education, and communication (IDREC) are necessary in the advancement of science educational perspectives from geophysics and climate research. In N. Callaos, E. Gaile-Sarkane, B. Sanchez, J. Horne and H.-W. Chu (Eds.), *WMSCI 2018 – 22^nd^ World Multi-Conference on Systemics, Cybernetics and Informatics, Proceedings, 3*, 238-247. IIIS.
97. Li H., Li S., Chen B., Xu C., Zhu J., and Du W. (2015). Research on ship wake acoustic imaging based on multi-beam sonar. *2014 Oceans – St. John’s, OCEANS 2014*. <https://doi.org/10.1109/OCEANS.2014.7003294>.
98. Li S., Sun H., and Yan L. (2011). A filtering method for generating DTM based on multi-scale mathematic morphology*. 2011 IEEE International Conference on Mechatronics and Automation,* 693-697. <https://doi.org/10.1109/ICMA.2011.5985745>.
99. Li T.-C., Hang H., Faloutsos M., and Efstathopoulos P. (2015). Trackadvisor: Taking back browsing privacy from third-party trackers. In Y. Liu and J. Mirkovic (Eds.), *Lecture Notes in Computer Science (including subseries Lecture Notes in Artificial Intelligence and Lecture Notes in Bioinformatics), 8995* (pp. 277-289). Springer. <https://doi.org/10.1007/978-3-319-15509-8_21>.
100. Li W., Chen Y., Hu H., and Tang C. (2020). See reference list for details.
101. Liciotti D., Ferroni G., Frontoni E., Squartini S., Principi E., Bonfigli R., Zingaretti P., and Piazza F. (2014). See reference list for details.
102. Liu X., Gönültas, E., and Studer C. (2018). Analog-to-feature (A2F) conversion for audio-event classification. *European Signal Processing Conference, 2018-September*, 2275-2279. <https://doi.org/10.23919/EUSIPCO.2018.8553060>.
103. Liu Z., Shin J., Xu Y., Winata G. I., Xu P., Madotto A., and Fung P. (2020). See reference list for details.
104. Lopatovska I., and Oropeza H. (2018). See reference list for details.
105. Lovato S. B., Piper A. M., and Wartella E. A. (2019). See reference list for details.
106. Malema H.K., and Musakwa W. (2016). Tweets and facebook posts, the novelty techniques in the creation of origin-destination models. In K. Brazdil, T. Cheng, W. Shi, X. Tong, Y. Liu, C. Pettit, V. Safar, S. Li, M. Madden, M. A. Brovelli, A. Coltekin, F. Anton, A. Stein and Q.-Q. Li, M. Sester, H. Kawashima, M. Tomkova, P. Rapant, K. HaeKyong, L. Halounova and M. A. Mostafavi (Eds.), *International Archives of the Photogrammetry, Remote Sensing and Spatial Information Sciences, 41,* 555-562. International Society for Photogrammetry and Remote Sensing. <https://doi.org/10.5194/isprsarchives-XLI-B2-555-2016>.
107. Malik K.M., Malik H., and Baumann R. (2019). See reference list for details.
108. Manikonda L., Deotale A., and Kambhampati S. (2018). What’s up with privacy? User preferences and privacy concerns in intelligent personal assistants. *Proceedings of the 2018 AAAI/ACM Conference on AI, Ethics, and Society*, 229–235. <https://doi.org/10.1145/3278721.3278773>.
109. Martin E. J. (2017). See reference list for details.
110. Masutani O., Nemoto S., and Hideshima Y. (2019). See reference list for details.
111. Matvienko G.G., and Sukhanov A.Y. (2014). Software system for simulation IPDA lidar sensing from space platform. In G. G. Matvienko and O. A. Romanovskii (Eds.), *Proceedings of SPIE – The International Society for Optical Engineering, 9292*. SPIE. <https://doi.org/10.1117/12.2075078>.
112. Mavropoulos T., Meditskos G., Symeonidis S., Kamateri E., Rousi M., and Tzimikas D., Papageorgiou L., Eleftheriadis C., Adamopoulos G., Vrochidis S., and Kompatsiaris I. (2019). See reference list for details.
113. McLean G., and Osei-Frimpong K. (2019). See reference list for details.
114. McNeill M., and Lyons D. (2019). A comparison of contextual bandit approaches to human-in-the-loop robot task completion with infrequent feedback. *2019 IEEE 31st International Conference on Tools with Artificial Intelligence (ICTAI)*, 117–124. <https://doi.org/10.1109/ICTAI.2019.00025>.
115. McReynolds E., Hubbard S., Lau T., Saraf A., Cakmak M., and Roesner F. (2017). See reference list for details.
116. Miao Y., Liu X., Choo K.-K. R., Deng R. H., Wu H., and Li H. (2019). Fair and dynamic data sharing framework in cloud-assisted internet of everything. *IEEE Internet of Things Journal*, *6*(4), 7201–7212. <https://doi.org/https://doi.org/10.1109/JIOT.2019.2915123>.
117. Miettinen M., and Sadeghi A.-R. (2018). Keynote: Internet of things or threats? On building trust in IoT. *2018 International Conference on Hardware/Software Codesign and System Synthesis*, Article 8525931. <https://doi.org/10.1109/CODESISSS.2018.8525931>.
118. Mirzamohammadi S., Chen J. A., Sani A. A., Mehrotra S., and Tsudik G. (2017). See reference list for details.
119. MobileHCI 2018 – Beyond Mobile: The Next 20 Years – 20^th^ International Conference on Human-Computer Interaction with Mobile Devices and Services, Conference Proceedings Adjunct. (2018). Association for Computing Machinery.
120. Mofrad M. H., and Mosse D. (2018). Speech recognition and voice separation for the internet of things. *ACM International Conference Proceeding Series,* Article a8. <https://doi.org/10.1145/3277593.3277610>.
121. Mokhtari M., de Marassé A., Kodys M., and Aloulou H. (2019). See reference list for details.
122. Mukherjee D., Kundu S., Kar T., and Chakraborty A. (2019). Controlling multiple Home appliances through Google assistant and monitoring sensor’s data from server. In S. Chakrabarti and A. Mukherjee (Eds.), I*EMECON 2019 – 9^th^ Annual Information Technology, Electromechanical Engineering and Microelectronics Conference*, (pp. 179-181), IEEE. <https://doi.org/10.1109/IEMECONX.2019.8876977>. .
123. Murad C. (2019). Tools to support voice user interface design. *Proceedings of the 21^st^ International Conference on Human-Computer Interaction with Mobile Devices and Services,* Article a72. <https://doi.org/10.1145/3338286.3344424>.
124. Murugesan S., and Balajiraja N. (2019). A study on various methods and application for examining the smart voice assistant device. *Journal of Advanced Research in Dynamical and Control Systems, 11*(12), 383-392, <https://doi.org/10.5373/JARDCS/V11SP12/20193234>.
125. Nesta F., Wada T. S., Miyabe S., and Juang B.-H. (2009). On the non-uniqueness problem and the semi-blind source separation. *IEEE Workshop on Applications of Signal Processing to Audio and Acoustics*, 101-104. <https://doi.org/10.1109/ASPAA.2009.5346539>.
126. Neto J.P., Cassaca R., Viveiros M., and Mourão M. (2006) Design of a multimodal input interface for a dialogue system. *Lecture Notes in Computer Science (including subseries Lecture Notes in Artificial Intelligence and Lecture Notes in Bioinformatics), 3960 LNAI*, 170-179. <https://doi.org/10.1007/11751984_18>.
127. Nhu T. V., and Sawada H. (2018). Development of Vietnamese Voice Chatbot with Emotion Expression. *MHS 2018 – 2018 29^th^ International Symposium on Micro-NanoMechatronics and Human Science,* Article 8886954. <https://doi.org/10.1109/MHS.2018.8886954>.
128. Nouri E., Sim R., Fourney A., and White R. W. (2020). Step-wise recommendation for complex task support. *Proceedings of the 2020 Conference on Human Information Interaction and Retrieval*, 203–212. <https://doi.org/10.1145/3343413.3377964>.
129. Oh S.-R., and Kim Y.-G. (2017). See reference list for details.
130. Ong D. T., De Jesus C. R., Gilig L. K., Alburo J. B., and Ong E. (2018). See reference list for details.
131. Ospan B., Khan N., Augusto J., Quinde M., and Nurgaliyev K. (2018). Context aware virtual assistant with case-based conflict resolution in multi-user smart home environment. *2018 International Conference on Computing and Network Communications (CoCoNet)*, 36–44. <https://doi.org/10.1109/CoCoNet.2018.8476898>.
132. Ozawa H. (2018). Developing artificial intelligence services that satisfy customer demands: Moving forward with social implementation of corevo® technologies. *NTT Technical Review, 16*(8), 7-11.
133. Palumbo F., Gallicchio C., Pucci R., and Micheli A. (2016). See reference list for details.
134. Parkin S., Patel T., Lopez-Neira I., and Tanczer L. (2019). See reference list for details.
135. Patel D., and Bhalodiya P. (2019). See reference list for details.
136. Pignotti A., Marcozzi D., Cifani S., Squartini S., and Piazza F. (2009). A blind source separation based approach for speech enhancement in noisy and reverberant environment. *Lecture Notes in Computer Science (including subseries Lecture Notes in Artificial Intelligence and Lecture Notes in Bioinformatics), 5641 LNAI,* 356-367. <https://doi.org/10.1007/978-3-642-03320-9_33>.
137. Pradhan A., Findlater L., and Lazar A. (2019). See reference list for details.
138. Pradhan A., Mehta K., and Findlater L. (2018). See reference list for details.
139. Pridmore J., and Mols A. (2020). See reference list for details.
140. Proceedings – 2015 2^nd^ International Conference on Mathematics and Computers in Sciences and in Industry, MCSI 2015. (2016). IEEE.
141. Purao S., and Meng C. (2019). See reference list for details.
142. Purington A., Taft J. G., Sannon S., Bazarova N. N., and Taylor S. H. (2017). See reference list for details.
143. Purohit N., Mane S., Soni T., Bhogle Y., and Chauhan G. (2019). A computer vision based smart mirror with virtual assistant. *2019 International Conference on Intelligent Computing and Control Systems,* 151-156. <https://doi.org/10.1109/ICCS45141.2019.9065793>.
144. Pyae A., and Joelsson T. N. (2018). See reference list for details.
145. Pyae A., and Scifleet P. (2019). See reference list for details.
146. Raj V., Chandran A., and Prabha A. (2019). IoT based smart home using multiple language voice commands*. 2019 2^nd^ International Conference on Intelligent Computing, Instrumentation and Control Technologies,* 1595-1599. <https://doi.org/10.1109/ICICICT46008.2019.8993202>.
147. Ramluckan T. (2019). Social media as a declaration of war? In L. Leenen, N. van der Waag-Cowling and N. van der Waag-Cowling (Eds.), *14^th^ International Conference on Cyber Warfare and Security, ICCWS 2019* (pp. 356-360). Academic Conferences and Publishing International Limited.
148. Ranoliya B.R., Raghuwanshi N., and Singh S. (2017). Chatbot for university related FAQs. *2017 International Conference on Advances in Computing, Communications and Informatics, ICACCI 2017, 2017-January*, 1525-1530. <https://doi.org/10.1109/ICACCI.2017.8126057>.
149. Reeves S., Porcheron M., and Fischer J. (2019). ‘This is not what we wanted’: Conversation designing with for voice interfaces. *Interactions, 26*(1), 47-51. <https://doi.org/10.1145/3296699>.
150. Ren J., Mandalari A.M., Dubois D.J., Kolcun R., Choffnes D., and Haddadi H. (2019). Information exposure from consumer IoT devices: A multidimensional, network-informed measurement approach. *Proceedings of the ACM SIGCOMM Internet Measurement Conference, IMC,* 267-279. <https://doi.org/10.1145/3355369.3355577>.
151. Robinson S., Pearson J., Ahire S., Ahirwar R., Bhikne B., Maravi N., and Jones M. (2018). See reference list for details.
152. Robledo-Arnuncio E., Wada T. S., and Juang B.-H. (2007). See reference list for details.
153. Saadaoui F. Z., Mahmoudi C., Maizate A., and Ouzzif M. (2019). See reference list for details.
154. Sakai Y., and Mitsuhashi W. (2008). A study on the property of blind source separation for preprocessing of an acoustic echo cancellar. *Proceedings of the SICE Annual Conference*, 13-18. <https://doi.org/10.1109/SICE.2008.4654614>.
155. Salazar A., Vergara L., Serrano A., and Igual J. (2010). A general procedure for learning mixtures of independent component analyzers. *Pattern Recognition, 43*(1), 69-85. <https://doi.org/10.1016/j.patcog.2009.05.013>.
156. Samarasinghe N., and Mannan M. (2019b). See reference list for details.
157. Samarasinghe N., and Mannan M. (2017). Short paper: TLS ecosystems in networked devices vs. web servers. In A. Kiayias (Ed.), *Lecture Notes in Computer Science (including subseries Lecture Notes in Artificial Intelligence and Lecture Notes in Bioinformatics), 10322 LNCS,* 533-541. Springer. <https://doi.org/10.1007/978-3-319-70972-7_30>.
158. Sanders J., and Martin-Hammond A. (2019). See reference list for details.
159. Sangal S., and Bathla R. (2019). See reference list for details.
160. Santhanaraj K., and Barkathunissa A. (2020). See reference list for details.
161. Santos-Pérez M., González-Parada E., and Cano-García J. M. (2011). See reference list for details.
162. Seymour W. (2018). See reference list for details.
163. Shamekhi A., Bickmore T., Lestoquoy A., and Gardiner P. (2017). See reference list for details.
164. Shayegh P., and Ghanavati S. (2017). Toward an approach to privacy notices in IoT. *Proceedings – 2017 IEEE 25^th^ International Requirements Engineering Conference Workshops, REW 2017,* 104-110. <https://doi.org/10.1109/REW.2017.77>.
165. Shaygan A., Haciane G., Lerdphayakkarat R., Kutch J. M., Roschke J., and Daim T. (2018). Decision Making Model for Choosing Voice-Operated Intelligent Speakers for Graduate Students. *2018 IEEE International Conference on Engineering, Technology and Innovation, ICE/ITMC 2018 – Proceedings*, Article 8436273. <https://doi.org/10.1109/ICE.2018.8436273>.
166. Shin C., Chandok P., Liu R., Nielson S.J., and Leschke T.R. (2018). See reference list for details.
167. Solorio J. A., Garcia-Bravo J. M., and Newell B. A. (2018). See reference list for details.
168. Souden M., and Liu Z. (2009). See reference list for details.
169. Srikanth S., Saddamhussain S. K., and Prasad P. S. (2019). See reference list for details.
170. Stadler S., Riegler S., and Hinterkörner S. (2012). Bzzzt: When mobile phones feel at home. *Conference on Human Factors in Computing Systems – Proceedings,* 1297-1302. <https://doi.org/10.1145/2212776.2212443>.
171. Stefanidi Z., Leonidis A., and Antona M. (2019). A multi-stage approach to facilitate interaction with intelligent environments via natural language. In C. Stephanidis and M. Antona (Eds.), *Communications in Computer and Information Science, 1088, (pp.* 67-77), Springer. <https://doi.org/10.1007/978-3-030-30712-7_9>.
172. Strzelecki A., and Rutecka P. (2020). Featured snippets results in Google web search: An exploratory study. In A. Rocha, J. L. Reis, M. K. Peter and Z. Bogdanovic (Eds.), *Smart Innovation, Systems and Technologies, 167,* (pp*.* 9-18). Springer. <https://doi.org/10.1007/978-981-15-1564-4_2>.
173. Sudharsan B., Corcoran P., and Ali M. I. (2019). See reference list for details.
174. Tao F., Liu G., and Zhao Q. (2018). See reference list for details.
175. Tarakji A.B., Xu J., Colmenares J.A., and Mohomed I. (2018). Voice enabling mobile applications with UIVoice*. EdgeSys 2018 – Proceedings of the 1^st^ ACM International Workshop on Edge Systems, Analytics and Networking, Part of MobiSys 2018*, 49-54. <https://doi.org/10.1145/3213344.3213353>.
176. Terzopoulos G., and Satratzemi M. (2019). Voice assistants and artificial intelligence in education. In G. Eleftherakis, M. Lazarova, A. Aleksieva-Petrova and A. Tasheva (Eds.), *ACM International Conference Proceeding Series*, Article a34. Association for Computing Machinery. <https://doi.org/10.1145/3351556.3351588>.
177. Thaha R., Jogi S. P., Rajan S., Mahajan V., Venugopal V. K., Mehndiratta A., and Singh A. (2020). Modified radial-search algorithm for segmentation of tibiofemoral cartilage in MR images of patients with subchondral lesion. *International Journal of Computer Assisted Radiology and Surgery, 15*(3), 403-413. <https://doi.org/10.1007/s11548-020-02116-z>.
178. Thapliyal H., Ratajczak N., Wendroth O., and Labrado C. (2018). See reference list for details.
179. Tielman M.L., Neerincx M.A., Bidarra R., Kybartas B., and Brinkman W.-P. (2017). See reference list for details.
180. Trimby M., and Pilgrim L. (2017). The impact of voice user interfaces. *Journal of the Institute of Telecommunications Professionals, 11,* 23-27.
181. Tsiourti C., Ben Moussa M., Quintas J., Loke B., Jochem I., Lopes J.A., and Konstantas D. (2018b). See reference list for details.
182. Tsiourti C., Quintas J., Ben-Moussa M., Hanke S., Nijdam N.A., and Konstantas D. (2018a). See reference list for details.
183. 2005 International Conference on Intelligent User Interfaces. (2005). In J. Riedl, A. Jameson, D. Billsus, T. Lau (Eds.), *Proceedings IUI.*
184. 2019 5^th^ International Conference on Mobile and Secure Services. (2019). In S. Piramuthu and P. Urien (Eds.), IEEE.
185. 2019 IEEE SoutheastCon. (2019), *Conference Proceedings – IEEE SOUTHEASTCON 2019*-*April,* IEEE.
186. Vaca K., Gajjar A., and Yang X.(2019). See reference list for details.
187. Victoire A. A. T., Gobu B., Jaikumar S., Arulmozhi N., Kanimozhi P., and Victoire A. T. (2019). Two-Stage Machine Learning Framework for Simultaneous Forecasting of Price-Load in the Smart Grid. In M. A. Wani, M. Sayed-Mouchaweh, E. Lughofer, J. Gama and M. Kantardzic (Eds.), *Proceedings – 17^th^ IEEE International Conference on Machine Learning and Applications, ICMLA 2018,* (pp. 1081-1086). IEEE. <https://doi.org/10.1109/ICMLA.2018.00176>.
188. Vishwakarma S. K., Upadhyaya P., Kumari B., and Mishra A. K. (2019). See reference list for details.
189. Vtyurina A., and Fourney A. (2018). Exploring the role of conversational cues in guided task support with virtual assistants. *Conference on Human Factors in Computing Systems – Proceedings, 2018-April*. <https://doi.org/10.1145/3173574.3173782>.
190. Wallace T., and Morris J. (2018). See reference list for details.
191. Wang Y., Kjerstad E., and Belisario B. (2020). A Dynamic Analysis Security Testing Infrastructure for Internet of Things. In P. Urien and S. Piramuthu (Eds.), *2020 6^th^ International Conference on Mobile and Secure Services,* Article 9042949. IEEE. <https://doi.org/10.1109/MobiSecServ48690.2020.9042949>.
192. Xue S., Yan Z., Yu T., and Liu Z. A. (2019). Study on Improving Acoustic Model for Robust and Far-Field Speech Recognition. *International Conference on Digital Signal Processing, DSP, 2018-November*, Article 8631862. <https://doi.org/10.1109/ICDSP.2018.8631862>.
193. Yaghoubzadeh R., Pitsch K., and Kopp S. (2015). See reference list for details.
194. Yildirim I., Bostanci E., and Guzel M. S. (2019). See reference list for details.
195. Yoneoka N., Arakawa Y., and Yasumoto K. (2019). Detecting Surrounding Users by Reverberation Analysis with a Smart Speaker and Microphone Array. *2019 IEEE International Conference on Pervasive Computing and Communications Workshops,* 523-528. <https://doi.org/10.1109/PERCOMW.2019.8730674>.
196. Yue C.Z., and Ping S. (2017). Voice activated smart home design and implementation. *Proceedings of 2017 2^nd^ International Conference on Frontiers of Sensors Technologies, 2017-January,* 489-492. <https://doi.org/10.1109/ICFST.2017.8210563>.
197. Zschörnig T., Wehlitz R., and Franczyk B. (2019). See reference list for details.

**B. Social Science domain (52)**

1. Akasaki S., and Kaji N. (2017). Chat detection in an intelligent assistant: Combining task-oriented and non-task-oriented spoken dialogue systems. *ACL 2017 – 55^th^ Annual Meeting of the Association for Computational Linguistics, Proceedings of the Conference (Long Papers)*, *1*, 1308-1319. <https://doi.org/10.18653/v1/P17-1120>.
2. Beirl D., Rogers Y., and Yuill N. (2019). Using voice assistant skills in family life. In K. Lund, G. P. Niccolai, E. Lavoue, C. Hmelo-Silver, G. Gweon and M. Baker. *Computer-Supported Collaborative Learning Conference, CSCL, 1*, 96-103. International Society of the Learning Sciences (ISLS).
3. Brasser F., Frassetto T., Riedhammer K., Sadeghi A.-R., Schneider T., and Weinert C. (2018). See reference list for details.
4. Brause S.R., and Blank G. (2020). See reference list for details.
5. Buchner H., and Kellermann W. (2008). A fundamental relation between blind and supervised adaptive filtering illustrated for blind source separation and acoustic echo cancellation. *2008 Hands-Free Speech Communication and Microphone Arrays, Proceedings*, 17–20. <https://doi.org/https://doi.org/10.1109/HSCMA.2008.4538677>.
6. Calaça J., Nobrega L., and Baras K. (2019). See reference list for details.
7. Dale R. (2017). The pros and cons of listening devices. *Natural Language Engineering, 23*(6), 969-973. <https://doi.org/10.1017/S1351324917000353>.
8. Druga S., Breazeal C., Williams R., and Resnick M. (2017). See reference list for details.
9. Dunin-Underwood A. (2020). See reference list for details.
10. Hamill L. (2006). See reference list for details.
11. Goud N., and Sivakami A. (2019). See reference list for details.
12. Herbordt W. (2005). 1 Introduction. In *Sound Capture for Human/Machine Interfaces: Lecture Notes in Control and Information Sciences, 315* (pp. 1-4). Springer. <https://doi.org/10.1007/11311942_1>.
13. Herron J. (2017). Intelligent Agents for the Library*. Journal of Electronic Resources in Medical Libraries, 14(3-4),* 139-144. <https://doi.org/10.1080/15424065.2017.1367633>.
14. Hoy M.B. (2018). See reference list for details.
15. Huxohl T., Pohling M., Carlmeyer B., Wrede B., and Hermann T. (2019). See reference list for details.
16. Jones V. K. (2018). See reference list for details.
17. Kandlhofer M., Steinbauer G., Hirschmugl-Gaisch S., and Huber P. (2016). See reference list for details.
18. King D. (2018). Hey, siri, what is the future of extension? *Journal of Extension, 56(5),* Article #5ED1.
19. King B., Chen I., Vaizman Y., Liu Y., Maas R., Parhasarathi S. H. K., and Hoffmeister B. (2017). See reference list for details.
20. Kita T., Nagaoka C., Hiraoka N., Suzuki K., and Dougiamas M. (2018). A discussion on effective implementation and prototyping of voice user interfaces for learning activities on moodle. In S. Zvacek, J. Uhomoibhi, B. M. McLaren and R. Reilly (Eds.), *CSEDU 2018 – Proceedings of the 10^th^ International Conference on Computer Supported Education, 1* (pp. 398-404). SciTePress.
21. Kita T., Nagaoka C., Hiraoka N., Suzuki K., and Dougiamas M. (2019). See reference list for details.
22. Kodali R. K., Rajanarayanan S. C., Boppana L., Sharma S., and Kumar A. (2019). See reference list for details.
23. Kumar A. (2018). See reference list for details.
24. Kuruvilla R. (2019). See reference list for details.
25. Lau J., Zimmerman B., and Schaub F. (2018). See reference list for details.
26. Li Y.-P., Yang J., Li X.-D., and Tian J. (2006). Ultrasonic intruder detection system for home security. In D.-S. Huang, Li K., Irwin G.W. (Eds*.), Intelligent Control and Automation.* *Lecture Notes in Control and Information Sciences, 344* (pp. 1108-1115). Springer. <https://doi.org/10.1007/11816492_143>.
27. Lopatovska I., and Oropeza H. (2018). See reference list for details.
28. Lopatovska I., Rink K., Knight I. Raines K., Cosenza K., Williams H., Sorsche P., Hirsch D., Li Q., and Martinez A. (2019). See reference list for details.
29. Maalsen S., and Sadowski J. (2019). The smart home on FIRE: Amplifying and accelerating domestic surveillance. *Surveillance and Society, 17*(1-2), 118-124. <https://doi.org/10.24908/ss.v17i1/2.12925>.
30. Martin E.J. (2017). See reference list for details.
31. Mc Lean G., and Osei-Frimpong K. (2019). See reference list for details.
32. Mtshali P., and Khubisa F. (2019). A smart home appliance control system for physically disabled people. *2019 Conference on Information Communications Technology and Society, ICTAS 2019,* Article 8703637. <https://doi.org/10.1109/ICTAS.2019.8703637>.
33. Nijholt A. (2006). Towards the automatic generation of virtual presenter agents. *Informing Science, 9,* 97-109.
34. Oh S.-R., Kim Y.-G. (2017). See reference list for details.
35. Ong D.T., De Jesus C.R., Gilig L.K., Alburo J.B., and Ong E. (2018). See reference list for details.
36. Pfeifle A. (2018). See reference list for details.
37. Pradhan A., Findlater L., Lazar A. (2019). See reference list for details.
38. Pridmore J., and Mols A. (2020). See reference list for details.
39. Pridmore J., Vitak J., Trottier D., Liao Y., Zimmer M., Mols A., and Kumar P.C. (2019). Intelligent personal assistants and the intercultural negotiations of dataveillance in platformed households. *Surveillance and Society, 17*(1-2), 125-131. <https://doi.org/10.24908/ss.v17i1/2.12936>.
40. Principi E., Squartini S., Piazza F., Fuselli D., and Bonifazi M. (2013). See reference list for details.
41. Ram S. S., Kumar C., Nandhini K. M., and Jayakar S. A. (2019). Artificial intelligence based home automation system. *International Journal of Scientific and Technology Research, 8*(7), 145-148.
42. Sahu P., Singh S. K., and Kumar P. (2019). Challenges and issues in securing data privacy in IoT and connected devices. *Proceedings of the 2019 6^th^ International Conference on Computing for Sustainable Global Development, INDIACom 2019*, 665-670.
43. Samarasinghe N., and Mannan M. (2019b). See reference list for details.
44. Samarasinghe N., and Mannan M. (2019a). See reference list for details.
45. Schiller A., and McMahon J. (2019). Alexa, alert me when the revolution comes: Gender, affect, and labor in the age of home-based artificial intelligence. *New Political Science, 41*(2), 173-191. <https://doi.org/10.1080/07393148.2019.1595288>.
46. Shin C., Chandok P., Liu R., Nielson S.J., and Leshke T.R. (2018). See reference list for details.
47. Son Y., and Oh W. (2018). “Alexa, buy me a movie!”: How AI speakers reshape digital content consumption and preference. *International Conference on Information Systems 2018, ICIS 2018*.
48. Tironi A., Mainetti R., Pezzera M., Borghese N. A. (2019). See reference list for details.
49. Turner-Lee N. (2019). See reference list for details.
50. Vora J., Tanwar S., Tyagi S., Kumar N., and Rodrigues J. J. P. C. (2017). See reference list for details.
51. Wallace T., and Morris J. (2018). See reference list for details.
52. Yusri M. M., Kasim S., Hassan R., Abdullah Z., Ruslai H., Jahidin K., and Arshad M.S. (2017). See reference list for details.

**C. Business & Management domain (20)**

1. Academy of Management 2007 Annual Meeting: Doing Well by Doing Good. (2007). Academy of Management 2007 Annual Meeting: Doing Well by Doing Good, AOM 2007.
2. Burns M. B., and Igou A. (2019). See reference list for details.
3. Das S., Ye Du A., Gopal R., and Ramesh R. (2011). Risk management and optimal pricing in online storage grids. *Information Systems Research*. <https://doi.org/10.1287/isre.1100.0288>.
4. Deshpande N. G., and Itole D. A. (2019). See reference list for details.
5. Eagles P. F., Johnson P. A., Potwarka L. R., and Parent C. (2015). Travel distance classes for tourism destinations: A proposal from Ontario Provincial Park camping. *Journal of Ecotourism, 14*(1), 64-84.
6. Goud N., and Sivakami A. (2019). See reference list for details.
7. Guthrie C. P., and Nicholls C. M. (2015). The personal budget project: A practical introduction to financial literacy. *Journal of Accounting Education, 33*(2), 138-163.
8. Hamill L. (2006). See reference list for details.
9. Hashemi S. H., Williams K. Kholy A. E., Zitouni I., and Crook P. A. (2018). See reference list for details.
10. Jones V. K. (2018). See reference list for details.
11. Karutz H. (2002). In-house fair at Hess was well-received. *Betonwerk und Fertigteil-Technik*, *68*(10), 58-59.
12. Khajeheian D. (2018). Enterprise social media: Ethnographic research on communication in entrepreneurial teams. *International Journal of E-Services and Mobile Applications, 10*(1), 34-46.
13. Kowalczuk P. (2018). See reference list for details.
14. Lackes R., Siepermann M., and Vetter G. (2019). See reference list for details.
15. Merritt R. (2007). Powerline home net camps mount urgent bid for unity. *Electronic Engineering Times, 1497*, 1.
16. Portillo C. D., and Lituchy T. R. (2018). See reference list for details.
17. Ram S. S., Kumar C., Nandhini K. M., Jayakar S. A. (2019). Artificial intelligence based home automation system. *International Journal of Scientific and Technology Research, 8*(7), 145-148.
18. Shaygan A., Haciane G., Lerdphayakkarat R., Kutch J.M., Roschke J., and Daim T. (2018). Decision making model for choosing voice-operated intelligent speakers for graduate students. *2018 IEEE International Conference on Engineering, Technology and Innovation (ICE/ITMC)*, (pp. 1-9). IEEE. <https://doi.org/10.1109/ICE.2018.8436273>.
19. Vishwakarma S. K., Upadhyaya P., Kumari B., and Mishra A. K. (2019). See reference list for details.
20. Wakefield C. C. (2019). See reference list for details.
